# Bibliometric analysis of slope-correcting osteotomy in the context of anterior cruciate ligament reconstruction: key trends, contributors and themes

**DOI:** 10.1007/s00590-026-05019-y

**Published:** 2026-10-27

**Authors:** Anish Loharuka, Nafisa Zilani, Siddarth Raj, Dhiren Babbar, Hasan Raza Mohammad, Hamid Razak, Matthieu Ollivier

**Affiliations:** 1 Queen Mary University of London, London, UK; 2 Centre for Bone and Joint Health, Queen Mary University of London, Blizard Institute, London, UK; 3 Guy‘s and St Thomas‘ NHS Foundation Trust, London, UK; 4 King’s Clinical Academic Training Office (KCATO), London, UK; 5 Centre for Bone and Joint Health, Queen Mary University of London, Blizard Institute, London, UK; 6 Department of Trauma and Orthopaedics, Royal London Hospital, London, UK; 7 Great Ormond Street Hospital, London, UK; 8 Total Orthopaedic care and surgery, Singapore, Singapore; 9 Hôpital Sainte-Marguerite, Marseille, France; 10 Aix-Marseille Université, Marseille, France

**Keywords:** Bibliometrics, Osteotomy, Posterior tibial slope, ACL reconstruction, Graft failure

## Abstract

**Purpose:**

Posterior tibial slope (PTS) is a key risk factor for anterior cruciate ligament (ACL) graft failure, prompting interest in slope-correcting osteotomy alongside ACL reconstruction (ACLR). This study mapped the literature on tibial osteotomy in relation to PTS and the ACL, a topic that, to the authors’ knowledge, has not previously been analysed bibliometrically.

**Methods:**

The Web of Science Core Collection was searched on 15 March 2026 following the BIBLIO guideline. Publication trends, productivity, citations, thematic maps and co-citation networks were analysed with bibliometrix (v5.2.1), with a sensitivity analysis restricted to original research articles.

**Results:**

Of 174 records, 151 were included (1993–2026): 73 original research articles, 42 reviews, 20 technique reports and 16 editorials or letters. Output rose from 10 publications in 2019 to 32 in 2025. The American Journal of Sports Medicine was the most productive and most cited journal (*n* = 24; 1114 citations). Authors from 29 countries contributed, led by the USA (*n* = 66), France (*n* = 39) and Germany (*n* = 30); collaborative publications were counted for each participating country. Co-citation analysis identified three interconnected clusters, and tibial deflexion osteotomy with ACLR was the principal motor theme. When the analysis was restricted to original research, nine of the 11 most cited publications remained among the 10 most cited.

**Conclusion:**

Research on slope-correcting osteotomy in ACLR has grown rapidly and is concentrated in few journals and research groups. Highly cited work is predominantly biomechanical or retrospective, suggesting a need for standardised indications, validated PTS thresholds and prospective comparative studies.

**Supplementary Information:**

The online version contains supplementary material available at https://doi.org/10.1007/s00590-026-05019-y.

## Introduction

Anterior cruciate ligament (ACL) reconstruction (ACLR) is a commonly performed orthopaedic procedure worldwide [1], yet graft failure rates are a significant clinical concern, with a rate of up to 34% in high-risk cohorts [2, 3]. Among the recognised risk factors for ACLR failure, posterior tibial slope (PTS) has emerged as a key anatomical variable, with meta-analytic evidence indicating that elevated PTS is associated with ACLR failure [4]. A PTS greater than 12° has been reported as a risk factor for ACL injury [5].

In response to this, slope-reducing anterior closing wedge high tibial osteotomy (ACWHTO) has gained considerable traction as a surgical adjunct to revision ACLR in patients with high tibial slopes [6, 7]. ACWHTO reduces the PTS, which might decrease the anterior force on the tibia, creating a more favourable mechanical environment for the ACL graft to function [8]. A recent meta-analysis reported improved knee stability, pivot-shift grade and subjective function after slope-reducing tibial osteotomy combined with primary or revision ACLR, with a low graft re-rupture rate, although direct comparisons with isolated ACLR remain scarce [9]. The long-term outcomes of combined ACLR and PTS correction are promising, with recent 20-year retrospective data suggesting durable stability and functional improvements in appropriately selected patients [10]. Bibliometric analysis offers a quantitative methodology to map the structure of a research field, identifying the most influential publications, prolific authors, leading institutions and evolving thematic trends over time. To the authors’ knowledge, no previous study has specifically analysed the bibliometric profile of osteotomy in relation to PTS in the context of ACLR. Therefore, the aim of this study was to perform a bibliometric analysis of the literature addressing tibial osteotomy in relation to PTS and the ACL, in order to characterise publication trends, identify key contributors and delineate research themes within this field. The bibliometric universe was thus defined as publications at the intersection of three concepts: tibial osteotomy, PTS and the ACL. Publications examining PTS as a risk factor for ACL injury or graft failure were included only when they explicitly addressed osteotomy as a means of correcting the slope; those without reference to osteotomy were outside the scope of this analysis.

## Methods

### Search strategy

This bibliometric analysis was conducted and reported in accordance with the preliminary guideline for reporting bibliometric reviews of the biomedical literature (BIBLIO) [11]. The Web of Science (WoS) Core Collection [12] was searched on 15 March 2026. WoS Core Collection was selected because it provides the complete cited-reference and citation-count fields required for the citation and co-citation analyses performed in bibliometrix. The search combined three concept blocks with the Boolean operator AND: (1) tibial osteotomy, (2) posterior tibial slope and (3) the ACL. Within each block, synonyms and alternative terms were combined with OR, and truncation (*) was used to capture variant word endings (e.g. osteotomy/osteotomies, reduction/reducing). To capture alternative terminology for slope correction, the osteotomy block contained both generic terms (tibial, high tibial and proximal tibial osteotomy) and procedure-specific terms (closing wedge, anterior closing wedge and deflexion osteotomy), and the slope block contained both anatomical terms (posterior tibial slope, tibial slope, sagittal slope) and procedural terms (slope correction, slope reduction, slope decrease). Because generic phrases such as ‘tibial osteotom*’ are contained within most procedure-specific names (e.g. slope-reducing high tibial osteotomy, tubercle-sparing anterior closing wedge proximal tibial osteotomy), publications using less common procedural names were retrieved provided they also referred to tibial slope and the ACL. Among the 174 retrieved records, publications describing supratuberosity or supratubercular (*n* = 14), infratuberosity or infratubercular (*n* = 14), tubercle-sparing or tubercle-preserving (*n* = 3), slope-changing or slope-modifying (*n* = 7) and deflexion or deflection (*n* = 20) osteotomies were captured because they also used a generic osteotomy term; publications using only less common procedural names, without any of the search terms, would not have been retrieved. The search was applied to the WoS Topic field (TS), which covers the title, abstract, author keywords and Keywords Plus. The exact executable search string is provided in Appendix 1. No date, language or document-type restrictions were applied at the search stage. The search retrieved 174 records, which were exported as full records with cited references in plain-text format.

### Eligibility criteria

Publications were eligible if they addressed tibial osteotomy in relation to the posterior tibial slope and the ACL, whether in the ACL-deficient, ACL-reconstructed or revision setting or in terms of the effect of osteotomy on the native ACL. This encompassed clinical outcome studies of combined or staged osteotomy and ACLR, biomechanical and cadaveric studies of the effect of slope modification on the ACL or ACL graft, radiographic and surgical-planning studies of slope correction, studies of PTS change after coronal-plane osteotomy in relation to the ACL, surgical technique reports and reviews. Studies of PTS as a risk factor for ACL injury or graft failure were eligible when they explicitly addressed slope-correcting osteotomy, for example as the indicated treatment or in defining a threshold for it. Records in which osteotomy, PTS or the ACL was mentioned only incidentally (for example, high tibial osteotomy for medial compartment osteoarthritis in which ACL status was not a subject of the study) were excluded. All document types were eligible, and the document type of every included record was recorded so that its influence could be examined in a sensitivity analysis. Records available only as early access (published online ahead of print and not yet assigned to a journal issue) were excluded, because their publication year may change on final publication, which would affect the annual production and citation-rate analyses.

### Study selection

**Fig. 1:**
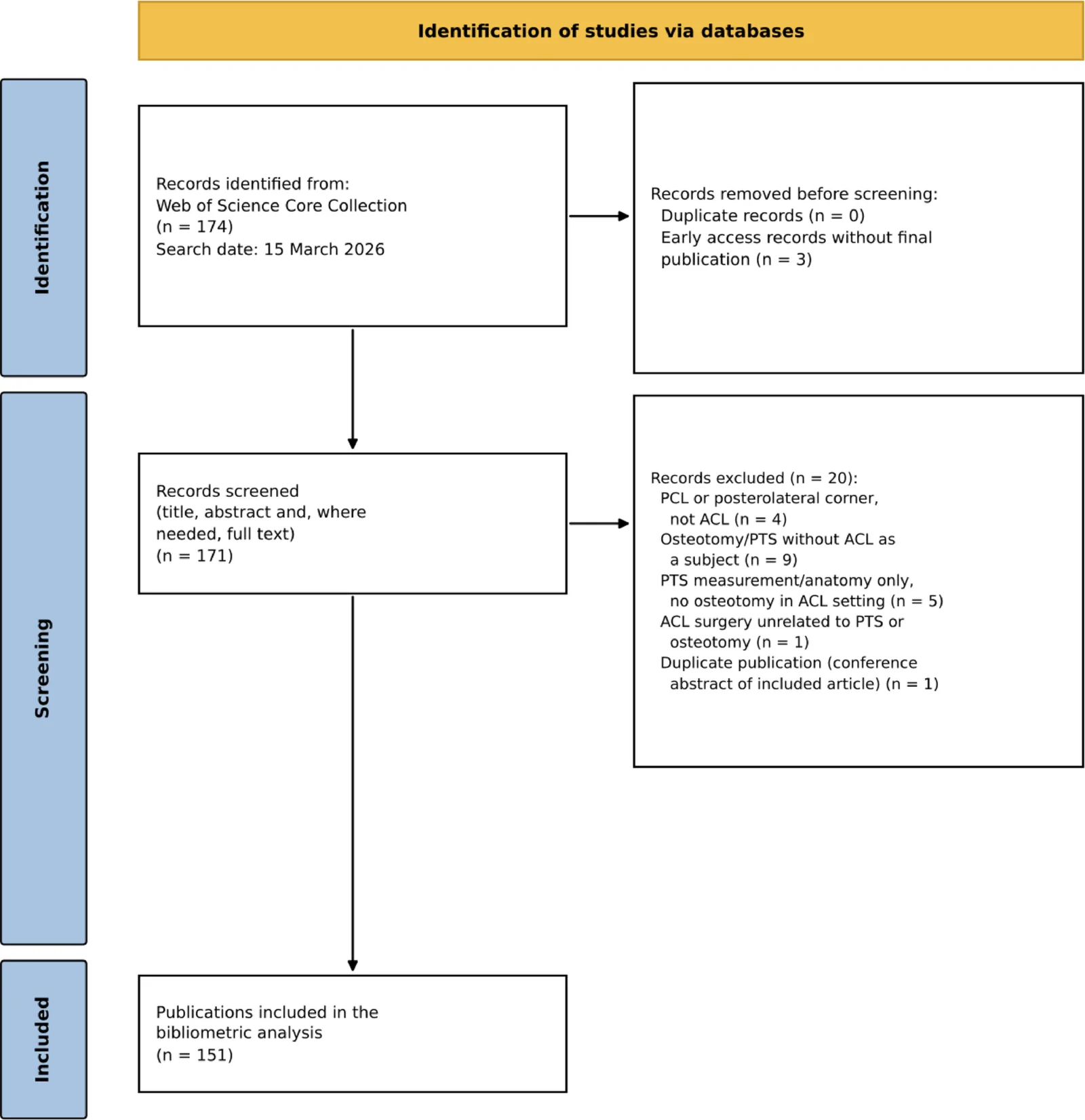
PRISMA 2020 flow diagram of the search and study selection process, with reasons for exclusion

Records were screened against the eligibility criteria by two authors (AL, NZ) working independently, using the title and abstract and consulting the full text where eligibility was unclear. Disagreements were resolved by discussion with a third author (SR). No formal inter-rater agreement statistic was calculated. Because a single database was searched, no duplicate records were expected; this was confirmed by checking WoS accession numbers and DOIs. The reason for each exclusion was recorded; excluded records are listed with reasons in Supplementary Table S2 and summarised in the PRISMA 2020 flow diagram (Fig. 1).

### Data extraction and analysis

Bibliometric analysis was performed on the included records using the bibliometrix package (version 5.2.1) [13] in R (version 4.5.3) via RStudio [14], with visualisations generated via the Biblioshiny graphical user interface [15]. Descriptive counts (document categories, journal, author, institution and country counts, and citation rankings) were tabulated directly from the exported WoS records using Python scripts, which are available from the authors. The following data fields were extracted from each record: title, abstract, document type, author details, author affiliations, source journal, year of publication, cited references and citation counts. Citation counts were those recorded in the WoS Core Collection on the date of the search. The bibliometric analysis was performed in accordance with the guidelines described by Donthu et al. [16]. The following analyses were performed. Annual scientific production was assessed to quantify the number of publications per year. Records with a 2026 publication year were retained in all analyses but are displayed separately in the annual production figure because 2026 was incomplete at the time of the search. The most productive authors, journals, institutions and countries were identified. Author name variants referring to the same individual were merged. Country and institution counts were calculated at the publication level using full counting: each publication was counted once for every country, and once for every institution, appearing in its author addresses; variant spellings of the same institution were merged, whereas a university and its affiliated hospital were counted separately. Internationally collaborative publications therefore contribute to more than one country, and country totals exceed the number of included publications. The country of the corresponding author was also recorded. Citation analysis was performed to identify the most influential publications, defined by total WoS Core Collection citation count; an annual citation rate (total citations divided by the number of years from the publication year to 2026, inclusive) was also calculated to partly offset the advantage of older publications. Keyword co-occurrence analysis, based on author keywords, was used to identify conceptual clusters. Thematic mapping positioned each cluster according to its centrality (the degree of interaction with other themes) and density (how strongly the keywords within a theme are connected to each other). Lastly, co-citation analysis was performed on the 30 most frequently co-cited references, measuring how often two references are cited together within the same publication. The network was restricted to 30 nodes to maintain visual interpretability. Thematic mapping used author keywords (250 words, minimum cluster frequency 5, three labels per cluster, Walktrap clustering); the trend topics analysis used author keywords with a minimum frequency of 5 and three words per year; and the co-citation network used the Fruchterman–Reingold layout with Walktrap clustering.

### Sensitivity analysis

Because document types differ in citation behaviour, the analyses most sensitive to document type were repeated after restricting the dataset to original research articles. Each included record was classified as an original research article (clinical, biomechanical/cadaveric or radiographic/imaging study), surgical technique report, review (narrative, systematic review or meta-analysis) or other (editorial, letter, correction or meeting abstract), using the WoS document-type field supplemented by the title, abstract and source journal (for example, WoS indexes technical notes in Arthroscopy Techniques and many systematic reviews as ‘Article’). Annual scientific production, journal productivity, citation analysis and thematic mapping were then repeated on the original research articles alone, using identical parameters.

## Results

### Search results

The search retrieved 174 records and no duplicate WoS records were identified. Three records published online as early access without a final publication were removed before screening. Of the 171 records screened, 20 were excluded: four addressed the posterior cruciate ligament or posterolateral corner rather than the ACL; nine studied osteotomy or PTS without the ACL as a subject (for example, high tibial osteotomy for osteoarthritis or genu recurvatum); five were PTS measurement or anatomical studies without reference to osteotomy in the ACL setting; one addressed ACL surgery unrelated to PTS or osteotomy; and one was a conference abstract duplicating an included full article. A total of 151 records were included (Fig. 1; Supplementary Table S2). The included corpus comprised 73 original research articles (39 clinical, 15 biomechanical or cadaveric and 19 radiographic, imaging or surgical-planning studies), 20 surgical technique reports, 42 reviews (8 systematic reviews or meta-analyses and 34 narrative reviews) and 16 editorials or letters (Table 1); all included publications are listed with their category in Supplementary Table S3. No randomised controlled trials were identified; clinical studies were predominantly retrospective case series and cohort studies (for example [17]). Six included records had a 2026 publication year; all had been assigned to a journal issue at the time of the search.

**Table 1:** Characteristics of the 151 included publications

Characteristic	*n* (%)
Original research articles	73 (48.3)
Clinical studies	39 (25.8)
Biomechanical or cadaveric studies	15 (9.9)
Radiographic, imaging or surgical-planning studies	19 (12.6)
Surgical technique reports	20 (13.2)
Reviews	42 (27.8)
Systematic reviews or meta-analyses	8 (5.3)
Narrative reviews	34 (22.5)
Editorials and letters	16 (10.6)
WoS document type: Article / Review / Editorial Material / Letter	112 / 24 / 13 / 2
Publication year 2026 (partial year)	6 (4.0)

Document categories were assigned from the WoS document type, title, abstract and source journal; WoS classifies technique reports and some reviews as ‘Article’. Percentages are of the 151 included publications

### Annual scientific production

**Fig. 2:**
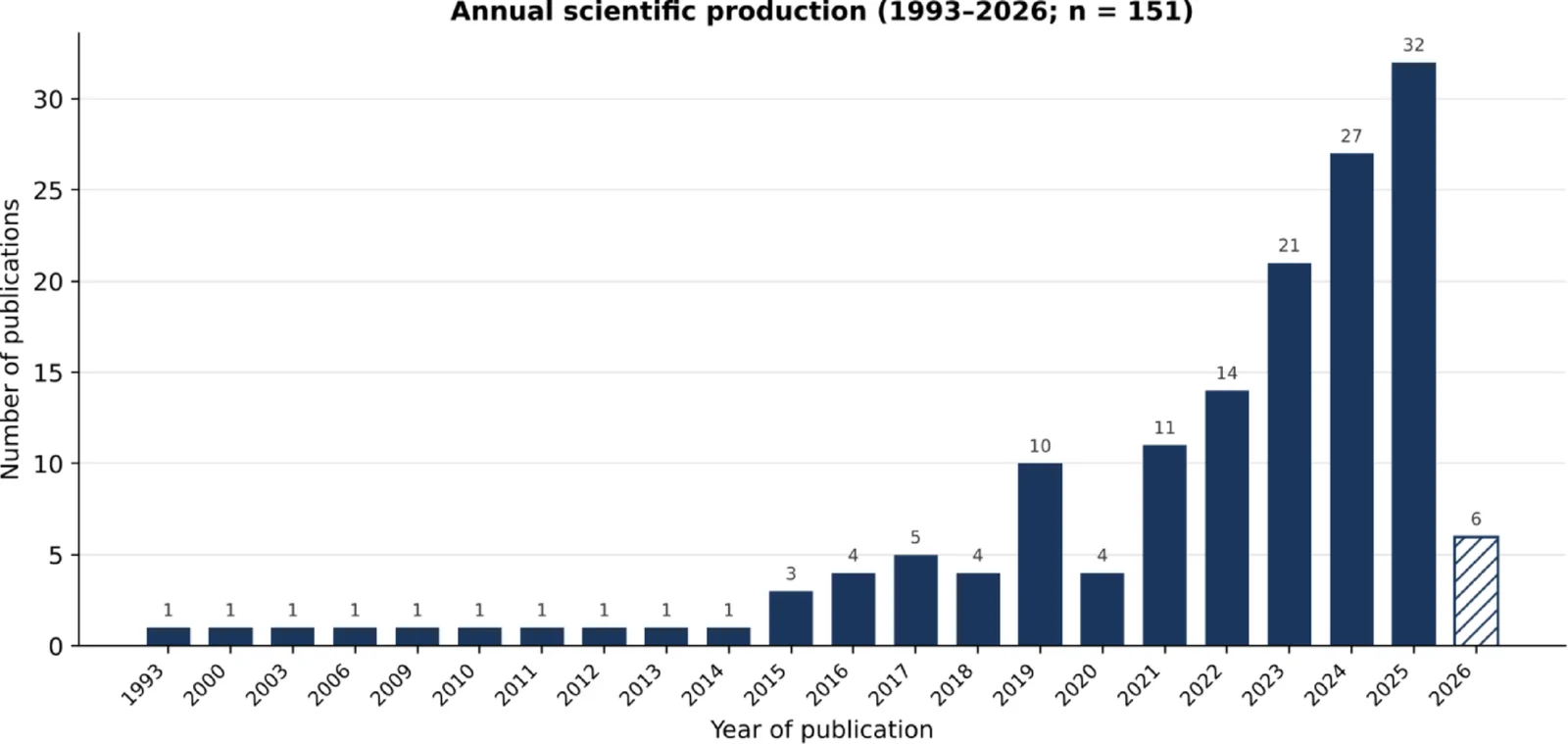
Annual scientific production, 1993–2026 (*n* = 151). The 2026 bar (hatched) represents a partial year, comprising records with a 2026 publication year retrieved up to 15 March 2026

The included publications spanned 1993–2026. Annual scientific production increased over the study period, with 129 of 151 publications (85.4%) appearing after 2017 (Fig. 2). Output rose from 10 publications in 2019 to 32 in 2025, and the five most recent complete years (2021–2025) accounted for 105 publications (69.5%). The six publications from 2026 are shown separately in Fig. 2 because 2026 was a partial year at the time of the search.

### Most productive journals

The 151 publications appeared in 42 journals, and were concentrated in a small number of specialist orthopaedic and sports medicine journals (Table 2).

**Table 2:** Journals with at least four included publications

Rank	Journal	Publications (*n*)	Citations (*n*)*
1	American Journal of Sports Medicine	24	1,114
2	Arthroscopy Techniques	17	71
3	Knee Surgery, Sports Traumatology, Arthroscopy	15	745
4	Arthroscopy: The Journal of Arthroscopic and Related Surgery	13	123
5	Journal of Experimental Orthopaedics	9	42
6	Orthopaedic Journal of Sports Medicine	7	177
7	Orthopaedics & Traumatology: Surgery & Research	6	63
8	Journal of ISAKOS	5	69
9	The Knee	4	35

*Total WoS Core Collection citations to the included publications in each journal. Seven further journals published three included publications each (Annals of Joint, Archives of Orthopaedic and Trauma Surgery, Arthroskopie, Clinics in Sports Medicine, Current Reviews in Musculoskeletal Medicine, EFORT Open Reviews and The Journal of Bone and Joint Surgery)

**Fig. 3:**
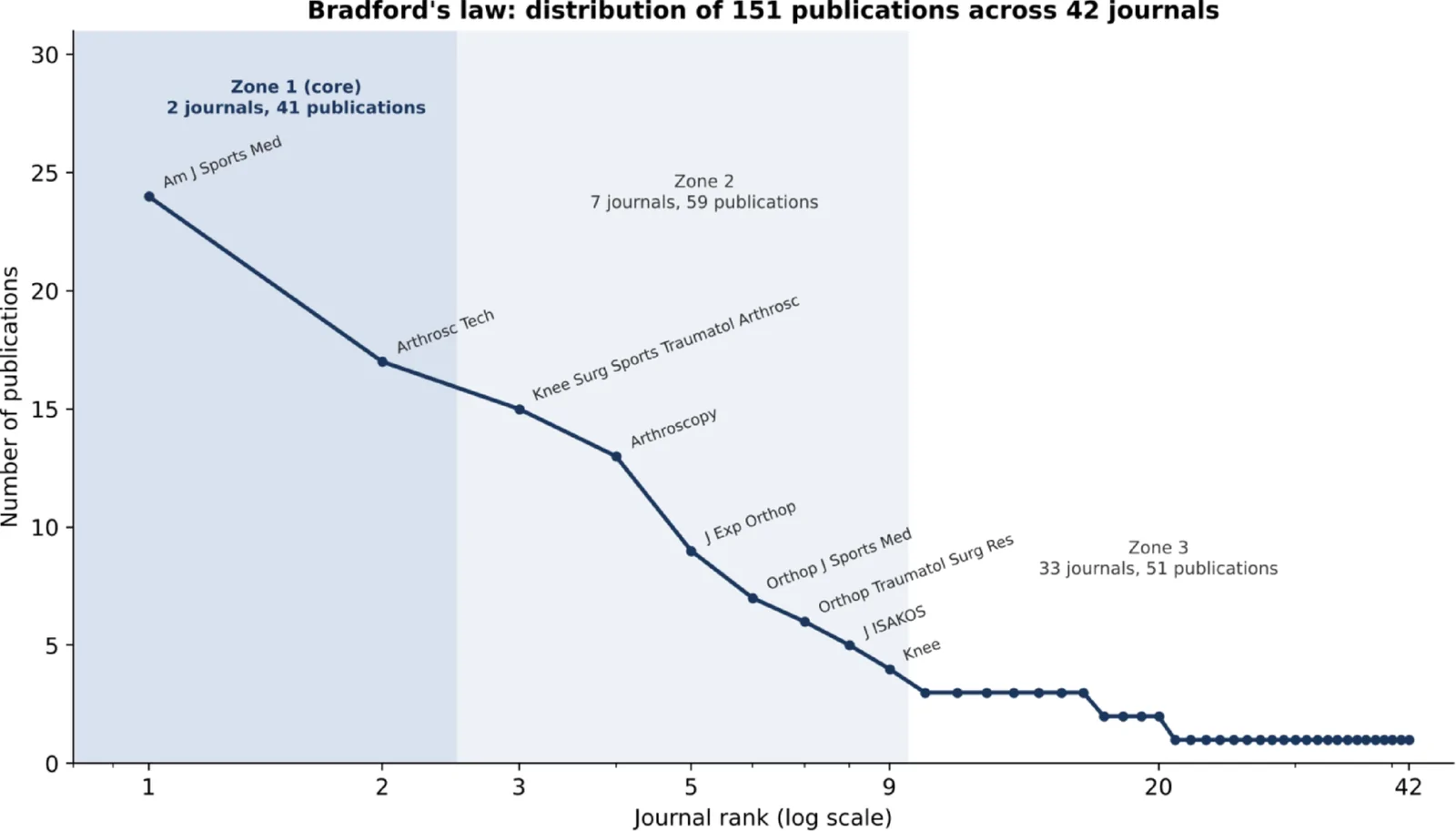
Bradford’s law of scattering. Zone 1 (core) comprised two journals, zone 2 seven journals and zone 3 33 journals, containing 27%, 39% and 34% of the 151 publications, respectively

The nine journals with at least four publications accounted for 100 of the 151 publications (66.2%). The American Journal of Sports Medicine was the most productive source (*n* = 24), followed by Arthroscopy Techniques (*n* = 17) and Knee Surgery, Sports Traumatology, Arthroscopy (*n* = 15) (Table 2). The American Journal of Sports Medicine also accumulated the most citations (1,114). Bradford’s law of scattering was applied to the results [18]. The distribution of publications was consistent with Bradford’s law: a core zone of two journals (The American Journal of Sports Medicine and Arthroscopy Techniques) contained 41 publications (27.2%), a second zone of seven journals contained 59 (39.1%), and the remaining 51 (33.8%) were scattered across 33 peripheral journals (Fig. 3).

### Authors, affiliations and countries analysis

The most published author among the included records was Matthieu Ollivier (Aix-Marseille University, France) with 18 publications, followed by David Dejour (Lyon Ortho Clinic, France) with 14 and Robert F. LaPrade (Twin Cities Orthopedics, USA) with 12 (Table 3).

**Table 3:** Most productive authors and local h-index

Rank	Author	Main affiliation, country	Publications (*n*)	Local h-index*
1	Matthieu Ollivier	Aix-Marseille University, France	18	8
2	David Dejour	Lyon Ortho Clinic/Clinique de la Sauvegarde, France	14	9
3	Robert F. LaPrade	Twin Cities Orthopedics, USA	12	7
4	Guillaume Demey	Lyon Ortho Clinic/Clinique de la Sauvegarde, France	9	7
4	Volker Musahl	University of Pittsburgh, USA	9	6
6	Shintaro Onishi	Aix-Marseille University, France/Hyogo Medical University, Japan	7	4
6	Luke V. Tollefson	Twin Cities Orthopedics, USA	7	3
8	Mo Saffarini	ReSurg SA, Switzerland	6	6
8	Nicolas Cance	Lyon Ortho Clinic, France	6	4
8	Christophe Jacquet	Aix-Marseille University, France	6	3

*h-index calculated within the included dataset from WoS Core Collection citation counts. Affiliations are those most frequently listed for the author in the included records. Name variants referring to the same author were merged (Ollivier M/MP; Dejour D/DH; LaPrade RF/RR; Tollefson L/LV)

**Table 4:** Institutions with at least seven included publications

Rank	Institution	Country	Publications (*n*)
1	Aix-Marseille University	France	16
2=	Assistance Publique-Hôpitaux de Marseille / Sainte-Marguerite Hospital	France	11
2=	Lyon Ortho Clinic / Clinique de la Sauvegarde	France	11
4=	Technical University of Munich	Germany	10
4=	University of Pittsburgh	USA	10
6=	Twin Cities Orthopedics	USA	9
6=	Steadman Philippon Research Institute / The Steadman Clinic	USA	9
8	University of Gothenburg / Sahlgrenska	Sweden	7

Publications with at least one author address at the institution, counted once per publication; variant spellings of the same institution were merged. A university and its affiliated hospital are listed separately and overlap. Five further institutions contributed six publications each (Centre Orthopédique Santy, Hôpital Privé Jean Mermoz, Hospital for Special Surgery, Hyogo Medical University and ReSurg SA)

Aix-Marseille University (*n* = 16) and Assistance Publique-Hôpitaux de Marseille/Sainte-Marguerite Hospital (*n* = 11) are a university and its affiliated hospital system and together represent the largest institutional contribution in this field, followed by Lyon Ortho Clinic/Clinique de la Sauvegarde (*n* = 11) (Table 4). Because many publications list both a university and its affiliated hospital, these counts overlap and should not be summed.

Authors from 29 countries contributed to the included publications. The USA contributed to the greatest number of publications (*n* = 66; 43.7%), followed by France (*n* = 39; 25.8%) and Germany (*n* = 30; 19.9%) (Table 5). Counts are at the publication level with full counting, so internationally collaborative publications contribute to each participating country; 64 publications (42.4%) involved authors from more than one country, which is why the country totals exceed 151. Address data were unavailable for seven publications. Ranked by corresponding-author country, the top three were unchanged (USA 50, France 23, Germany 20).

**Table 5:** Countries contributing to the included publications

Rank	Country	Publications (*n*)	% of 151	Corresponding author (*n*)
1	USA	66	43.7	50
2	France	39	25.8	23
3	Germany	30	19.9	20
4	Switzerland	11	7.3	9
5	China	10	6.6	8
5	Italy	10	6.6	5
5	United Kingdom	10	6.6	0
8	Japan	9	6.0	5
9	Australia	8	5.3	5
9	Korea	8	5.3	8
11	Sweden	7	4.6	0
12	Canada	6	4.0	2
13	Turkey	5	3.3	0
13	Austria	5	3.3	0
15	India	3	2.0	2
15	Chile	3	2.0	0
15	Spain	3	2.0	2

Full counting at the publication level: a publication is counted once for each country in its author addresses. Twelve further countries contributed to one or two publications each (Netherlands, Thailand and United Arab Emirates, 2 each; Argentina, Egypt, Luxembourg, Malaysia, Norway, Portugal, Qatar, Saudi Arabia and Singapore, 1 each). Percentages use all 151 included publications as the denominator; address data were unavailable for seven

### Most cited articles

Table 6 presents the 10 most cited publications (two publications were tied for tenth place); the 50 most cited publications, with document category, are listed in Supplementary Table S1.

**Table 6:** Top 10 most cited publications and annual citation rate

Rank	First author	Title	Journal	Year	Citations (WoS)	Annual citation rate*	Category
1	Dejour et al.	Tibial slope correction combined with second revision ACL produces good knee stability and prevents graft rupture	KSSTA	2015	238	19.8	Clinical
2	Shelburne et al.	Effect of Posterior Tibial Slope on Knee Biomechanics during Functional Activity	J Orthop Res	2011	214	13.4	Biomechanical
3	Bernhardson et al.	Tibial Slope and Its Effect on Force in Anterior Cruciate Ligament Grafts: Anterior Cruciate Ligament Force Increases Linearly as Posterior Tibial Slope Increases	AJSM	2019	209	26.1	Biomechanical
4	Sonnery-Cottet et al.	Proximal Tibial Anterior Closing Wedge Osteotomy in Repeat Revision of Anterior Cruciate Ligament Reconstruction	AJSM	2014	199	15.3	Clinical
5	Rodner et al.	Medial opening wedge tibial osteotomy and the sagittal plane - The effect of increasing tibial slope on tibiofemoral contact pressure	AJSM	2006	148	7.0	Biomechanical
6	Imhoff et al.	Slope-reducing tibial osteotomy decreases ACL-graft forces and anterior tibial translation under axial load	KSSTA	2019	123	15.4	Biomechanical
7	Song et al.	Slope-Reducing Tibial Osteotomy Combined With Primary Anterior Cruciate Ligament Reconstruction Produces Improved Knee Stability in Patients With Steep Posterior Tibial Slope, Excessive Anterior Tibial Subluxation in Extension, and Chronic Meniscal Posterior Horn Tears	AJSM	2020	90	12.9	Clinical
8	Yamaguchi et al.	Effects of Anterior Closing Wedge Tibial Osteotomy on Anterior Cruciate Ligament Force and Knee Kinematics	AJSM	2018	80	8.9	Biomechanical
9	Tischer et al.	The Impact of Osseous Malalignment and Realignment Procedures in Knee Ligament Surgery A Systematic Review of the Clinical Evidence	Orthop J Sports Med	2017	70	7.0	Systematic review
10=	Cantin et al.	The role of high tibial osteotomy in the treatment of knee laxity: a comprehensive review	KSSTA	2015	65	5.4	Narrative review
10=	Voos et al.	Effect of tibial slope on the stability of the anterior cruciate ligament-deficient knee	KSSTA	2012	65	4.3	Biomechanical

*Annual citation rate = total WoS Core Collection citations divided by the number of years from the year of publication to 2026, inclusive. AJSM, American Journal of Sports Medicine; KSSTA, Knee Surgery, Sports Traumatology, Arthroscopy; J Orthop Res, Journal of Orthopaedic Research; Orthop J Sports Med, Orthopaedic Journal of Sports Medicine

The most cited publication was “Tibial slope correction combined with second revision ACL produces good knee stability and prevents graft rupture” by Dejour et al., published in Knee Surgery, Sports Traumatology, Arthroscopy (KSSTA) [6], with 238 citations and an annual citation rate of 19.8. The publication with the highest annual citation rate was “Tibial Slope and Its Effect on Force in Anterior Cruciate Ligament Grafts” by Bernhardson et al. [19] (26.1 citations per year). Of the 11 publications in Table 6, nine were original research (six biomechanical or cadaveric and three clinical), one was a systematic review and one a narrative review. Together they accumulated 1,501 WoS Core Collection citations, and the 50 most cited publications accumulated 2,734.

### Sensitivity analysis restricted to original research articles

When the analysis was restricted to the 73 original research articles, the upward trend in output persisted (5 publications in 2019 and 15 in 2025; 46 [63.0%] in 2021–2025). The American Journal of Sports Medicine (*n* = 23) and Knee Surgery, Sports Traumatology, Arthroscopy (*n* = 13) remained the two most productive journals, whereas Arthroscopy Techniques no longer featured because its included publications were technique reports and one editorial. Nine of the 11 publications in Table 6 remained among the 10 most cited; the systematic review by Tischer et al. and the narrative review by Cantin et al. dropped out, and Zaffagnini et al. (2013) entered. In the thematic map of original research articles (Supplementary Fig. S1), posterior tibial slope, anterior cruciate ligament and high tibial osteotomy remained the largest basic theme, and anterior tibial closing wedge osteotomy with PTS remained a motor theme; the tibial deflexion osteotomy cluster moved to the boundary between the motor and niche quadrants, and knee stability with slope-reducing tibial osteotomy moved from the basic to the niche quadrant.

### Thematic mapping

**Fig. 4:**
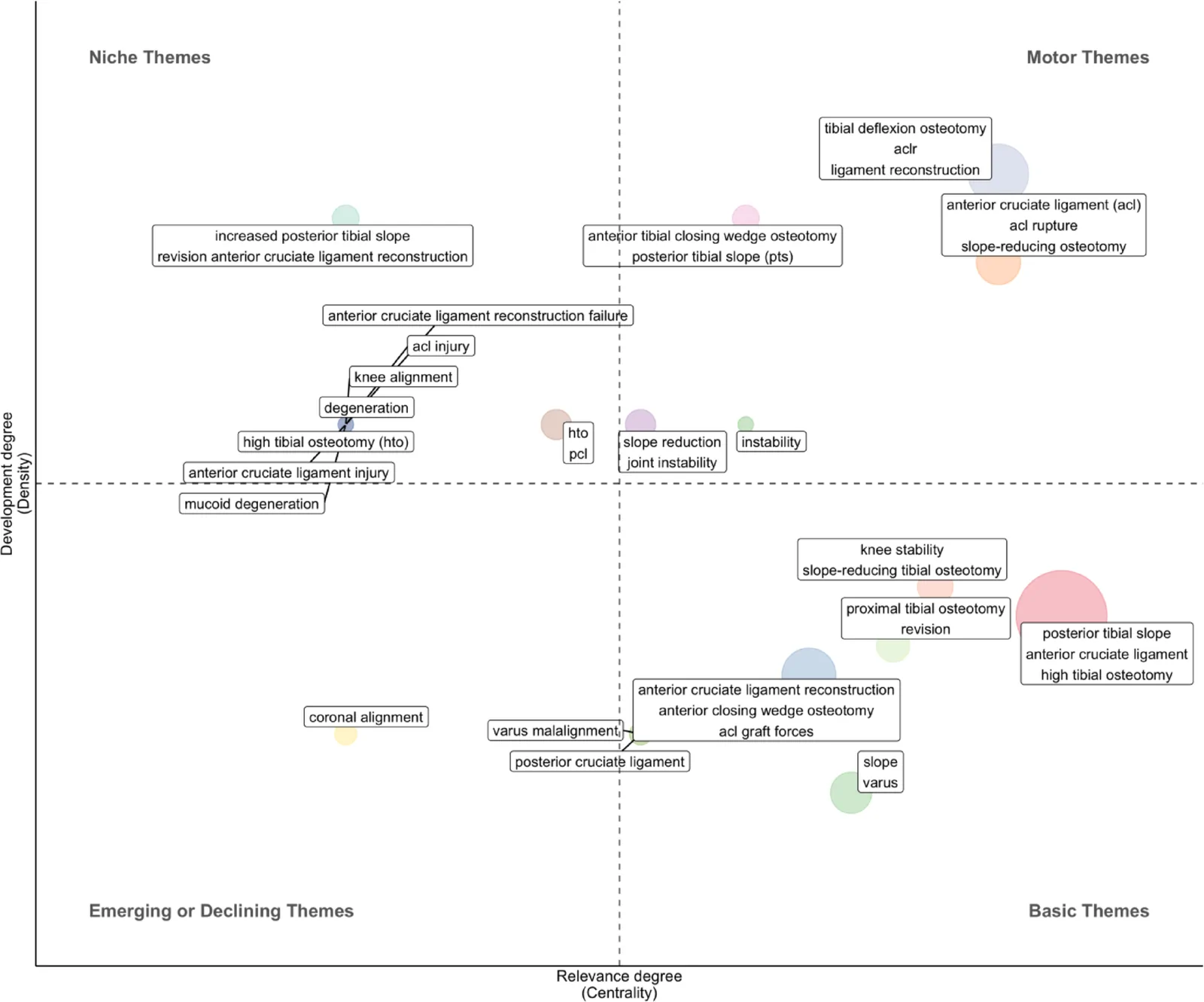
Thematic map of author keyword clusters (*n* = 151 publications), positioned by centrality (relevance) and density (development). Bubble size is proportional to keyword frequency

Thematic mapping based on author keyword co-occurrence identified clusters in all four quadrants (Fig. 4). Motor themes (high centrality, high density) were led by a cluster comprising tibial deflexion osteotomy, ACLR and ligament reconstruction, together with smaller clusters containing ACL rupture and slope-reducing osteotomy, and anterior tibial closing wedge osteotomy with PTS. Basic themes (high centrality, low density) were dominated by the largest cluster, comprising posterior tibial slope, anterior cruciate ligament and high tibial osteotomy, and also included knee stability with slope-reducing tibial osteotomy, proximal tibial osteotomy with revision, and ACL reconstruction with anterior closing wedge osteotomy and ACL graft forces. Niche themes (low centrality, high density) included increased posterior tibial slope with revision ACL reconstruction, and several small single-keyword clusters (for example ACL reconstruction failure, knee alignment and mucoid degeneration). Coronal alignment was the only cluster clearly located in the emerging or declining quadrant; varus malalignment and posterior cruciate ligament lay at the boundary between the emerging and basic quadrants. Smaller clusters not described here are shown in Fig. 4.

### Keyword trend analysis

**Fig. 5:**
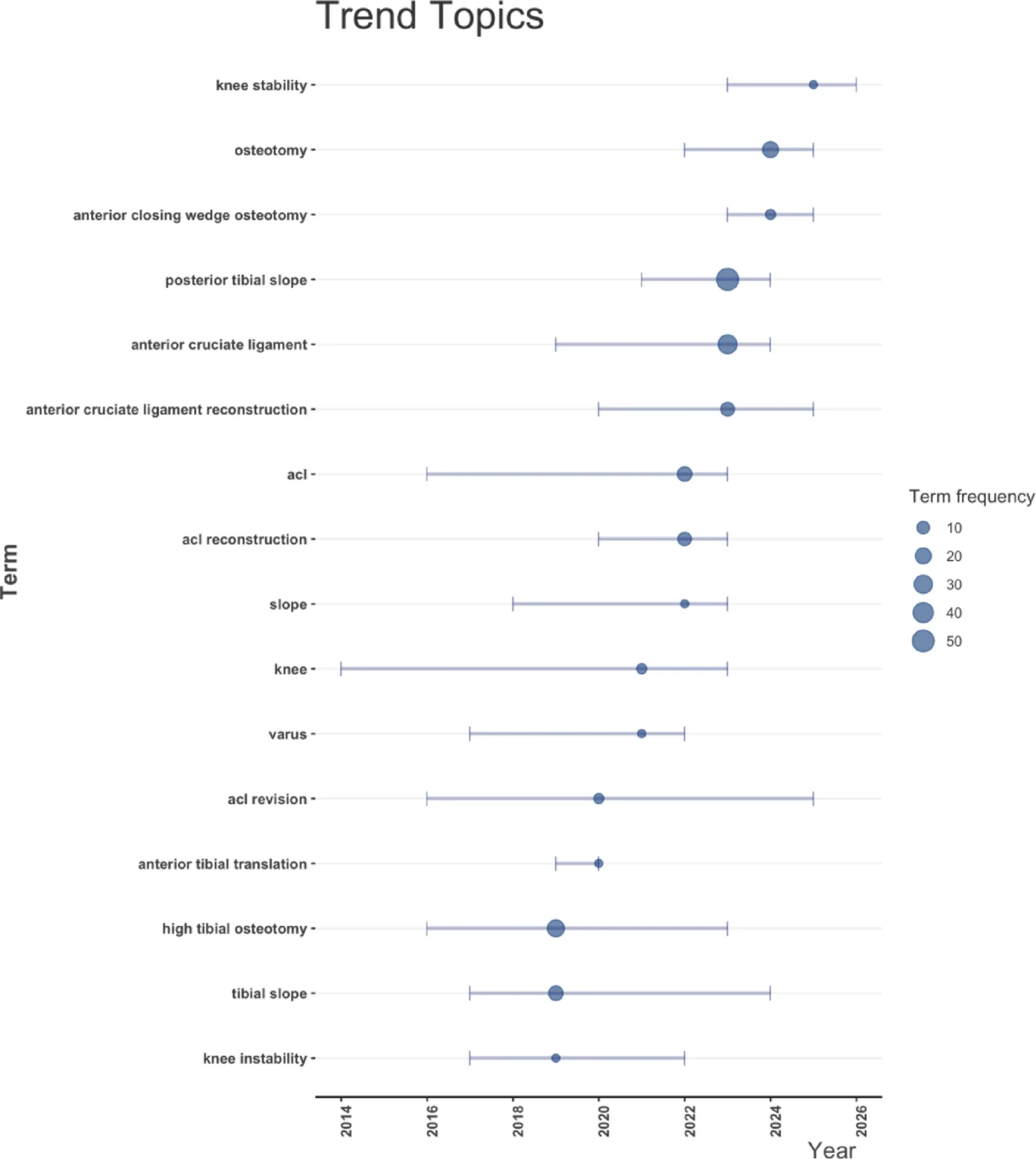
Trend topics analysis of author keywords (minimum frequency 5). Points show the median year of use and lines the interquartile range; point size is proportional to term frequency

Keyword trend analysis was performed to examine the temporal distribution of author keywords (Fig. 5). Posterior tibial slope and anterior cruciate ligament were the most frequent terms. High tibial osteotomy, tibial slope, knee instability and anterior tibial translation had median years of 2019–2020, whereas anterior closing wedge osteotomy, osteotomy and knee stability had the most recent median years (2024–2025).

### Co-citation network

**Fig. 6:**
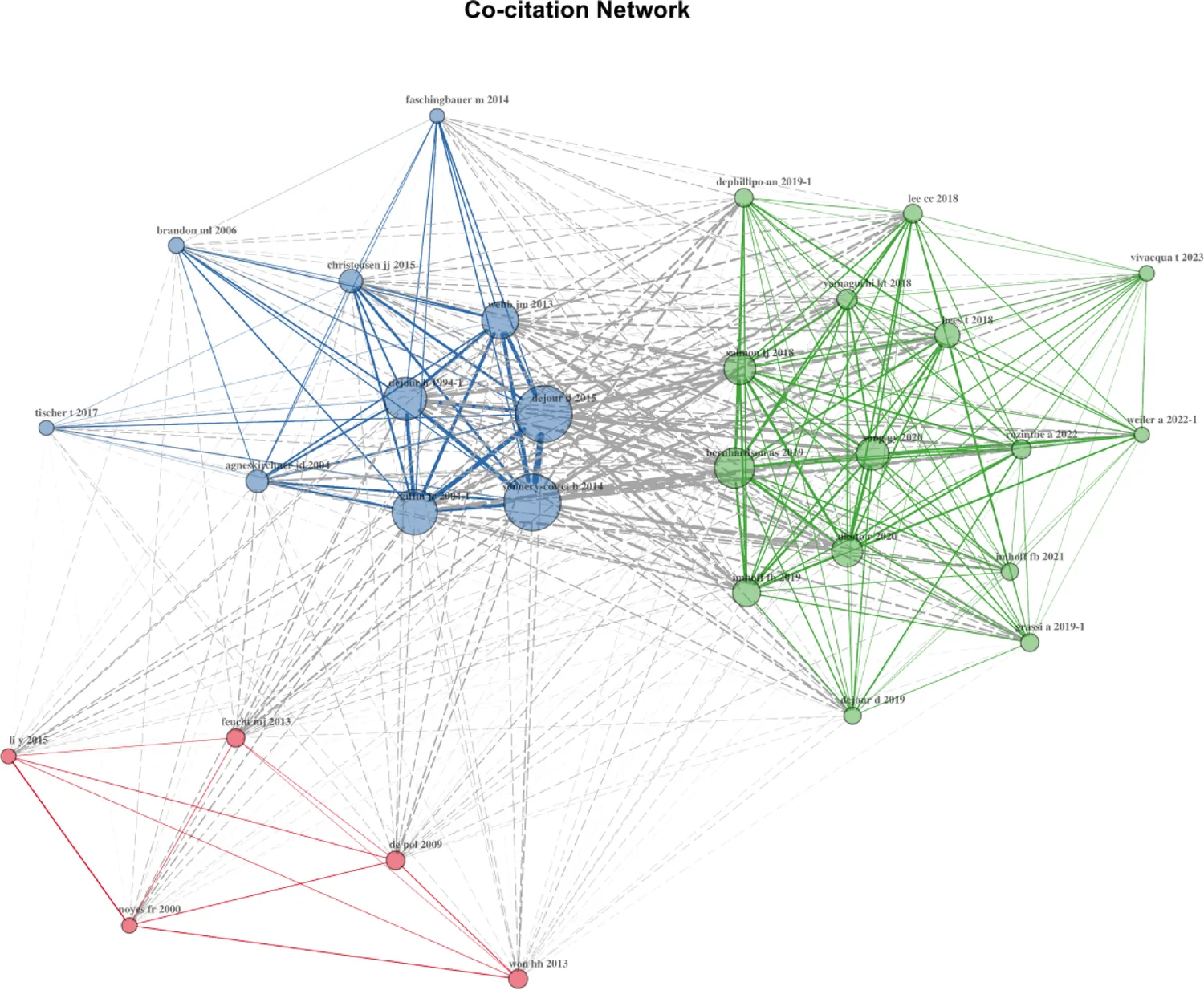
Co-citation network of the 30 most frequently co-cited references (Fruchterman–Reingold layout, Walktrap clustering). Node size reflects the number of co-citations; colours indicate clusters

Co-citation analysis was performed on the 30 most frequently co-cited references to identify the intellectual base of this literature. Network visualisation using the Fruchterman-Reingold algorithm [20] identified three clusters: two large, densely interlinked clusters and a smaller, more peripheral third cluster (Fig. 6).

The first cluster (10 references) contained early studies of tibial slope and the ACL together with the clinical series of slope correction combined with revision ACLR, including Dejour et al. [6] and Sonnery-Cottet et al. [7]. The second and largest cluster (15 references) contained more recent biomechanical studies of slope and ACL graft force, including Bernhardson et al. [19], Yamaguchi et al. [25] and Imhoff et al. [27], and clinical studies of slope-reducing osteotomy, including Song et al. [26]. The third, smaller cluster (5 references) concerned high tibial osteotomy technique and its effect on tibial slope. The third cluster was connected to the other two mainly by weaker links.

## Discussion

This bibliometric analysis maps the literature on slope-correcting osteotomy in the context of ACLR. The principal findings were: (1) publication output increased markedly after 2017, with 32 publications in 2025 compared with 10 in 2019; (2) publications were concentrated in a small number of specialist sports medicine and arthroscopy journals, consistent with Bradford’s law; (3) the USA, France and Germany contributed the most publications, and the most productive institution, author and most cited publication were French; (4) original research made up just under half of the corpus, the most cited publications were predominantly biomechanical and retrospective clinical studies, no randomised trials were identified, and the citation rankings were largely unchanged when the analysis was restricted to original research; and (5) co-citation analysis showed three clusters, two of them densely interlinked, and thematic mapping showed that tibial deflexion osteotomy combined with ACLR was the principal motor theme, with posterior tibial slope, the ACL and high tibial osteotomy forming the main basic theme.

The first key finding is the growth in publication output, most of which occurred after 2017. Bibliometric data describe this growth but cannot establish its cause. It coincides temporally with the publication of biomechanical and clinical studies linking increased PTS to greater ACL graft force, greater anterior tibial translation and graft failure [19, 21, 22], and with systematic reviews of high tibial osteotomy for knee instability [23]. Other plausible contributors include the introduction and refinement of anterior closing wedge and tubercle-sparing techniques, growth in revision ACLR volume and a general increase in orthopaedic publication output; the relative contribution of each cannot be determined from the present data.

The second key finding is the concentration of publications within a small number of journals. The American Journal of Sports Medicine, Arthroscopy Techniques and Knee Surgery, Sports Traumatology, Arthroscopy together published 56 of the 151 publications, and the distribution followed Bradford’s law. The core zone included Arthroscopy Techniques, reflecting the number of surgical technique reports in this field (20 of 151 publications); this journal dropped out of the rankings when only original research was considered. The American Journal of Sports Medicine and Knee Surgery, Sports Traumatology, Arthroscopy also published most of the highly cited studies.

The third key finding concerns the geographic and institutional distribution of research. The USA contributed to the most publications, but France was home to the most productive institution (Aix-Marseille University and its affiliated hospital system), the two most productive authors and the most cited publication, by Dejour et al. (2015), which reported good knee stability and no graft re-rupture after slope correction combined with second revision ACLR [6]. More than two-fifths of publications involved international collaboration. These observations describe where the literature is produced; they do not by themselves measure conceptual influence.

The fourth key finding relates to the composition of the most cited literature. Original research accounted for 73 of the 151 publications, with the remainder made up of reviews (42), surgical technique reports (20) and editorials or letters (16). Nine of the 11 most cited publications were original research, six of them biomechanical or cadaveric studies, and no randomised controlled trials were identified; clinical studies were predominantly retrospective case series and cohort studies. Restricting the analysis to original research removed the two reviews from the most cited list and the technique-focused journal from the journal rankings, but did not alter the publication trend or the leading journals. This pattern indicates that much of the highly cited evidence in this field derives from biomechanical analyses and retrospective clinical series, with few prospective comparative studies. It also illustrates why citation rankings that pool all document types should be interpreted with caution [24].

The fifth key finding is the intellectual structure of the field. Co-citation analysis identified three clusters, the two largest densely interlinked, indicating that publications draw on a largely shared set of foundational studies. One cluster combines early work on tibial slope and the ACL with the clinical series of slope correction [6, 7]; the largest combines biomechanical investigations of PTS and ACL graft force with recent clinical studies of slope-reducing osteotomy [19, 25, 26]; and a smaller cluster concerns high tibial osteotomy technique and slope change. The distribution of references across these clusters broadly follows their publication dates, suggesting a progression from describing the problem to evaluating and refining its surgical correction, although co-citation data cannot establish this directly. The field remains organised around the concept that sagittal-plane factors, including bony alignment and movement patterns, influence ACL loading [22, 27, 28].

Thematic mapping is consistent with this: tibial deflexion osteotomy with ACLR formed the principal motor theme, while posterior tibial slope, the ACL and high tibial osteotomy formed the largest basic theme, central to the field but less internally developed. Tibial deflexion osteotomy, anterior closing wedge osteotomy and slope-reducing osteotomy largely describe the same procedure, so their appearance in separate clusters partly reflects terminology rather than distinct research themes; ‘deflexion’ was used predominantly by French groups (14 of the 18 included publications using the term in the title, abstract or keywords had a French author address).

Several more peripheral themes are of interest. Bibliometric finding: increased posterior tibial slope with revision ACL reconstruction formed a niche cluster, and coronal alignment was located in the emerging or declining quadrant. Interpretation: the niche cluster is internally cohesive but weakly connected with the rest of the field, whereas coronal alignment may be either an emerging or a declining topic; its presence may reflect attention to the multiplanar effects of slope-correcting osteotomy. Supporting literature: PTS values differ according to the reference axis used for measurement (mechanical versus proximal anatomical axis), which affects intervention thresholds and comparability across studies [29]; combined assessment of PTS with static anterior tibial translation and other factors has been proposed in place of a single slope threshold [30, 31]; and recent clinical data suggest that lateral, rather than medial, PTS may be more strongly associated with early graft failure, although this remains unresolved [32]. Bibliometric finding: anterior closing wedge osteotomy and knee stability were the most recent trend topics. Supporting literature: a recent meta-analysis found improved stability after combined slope-reducing osteotomy and ACLR but noted that direct comparisons with isolated ACLR remain scarce [9]. These clinical observations are cited to contextualise the bibliometric findings and were not generated by the present analysis.

Taken together, the bibliometric profile describes a literature that is growing, interconnected and concentrated, but whose highly cited evidence base is dominated by biomechanical, retrospective and technical work. Clinical studies within the corpus, such as those by Dejour et al. and Song et al., report improved knee stability and low graft failure rates when slope correction is combined with ACLR [6, 26], but surgical indications, techniques (e.g. anterior closing wedge versus opening wedge osteotomy) and PTS measurement methods vary between studies, and the PTS threshold associated with graft rupture is still debated [33]. Bibliometric methods cannot determine whether slope-correcting osteotomy is clinically effective; they indicate where evidence has accumulated and where it remains sparse.

### Limitations

#### This study has several limitations

First, only the WoS Core Collection was searched, so relevant publications indexed solely in Scopus, PubMed or other databases may have been missed. WoS was chosen because it provides the cited-reference data required for co-citation analysis, and the use of a single database is shared by other bibliometric analyses [34–36]. Second, the search required osteotomy, slope and ACL terms to co-occur in the topic fields. Publications on PTS and ACL failure that did not refer to osteotomy were therefore excluded by design, and publications describing slope correction using terms not included in the search string may have been missed, although generic osteotomy terms were included to minimise this risk. Third, citation-based metrics are subject to recognised biases. Older publications accumulate citations over a longer period [37]; the annual citation rate only partly mitigates this, and very recent publications, including those from 2026, are under-represented in citation rankings. Self-citation was not excluded and may inflate the counts of highly productive groups. In addition, one of the authors of this study is among the most productive authors in the dataset; to limit bias, eligibility was decided against explicit criteria and all counts and rankings were derived directly from the WoS records. Fourth, the corpus contains heterogeneous document types, including reviews and technical reports, which differ in citation behaviour [24]. This was addressed with a sensitivity analysis restricted to original research articles, but classification of document type relied partly on WoS indexing. Fifth, country and institution counts used full counting of author affiliations, so multinational and multi-institutional publications contribute to each participating country and institution. This inflates the totals of highly collaborative groups, and related institutions (e.g. a university and its affiliated hospital system) were not merged and appear separately. Author keywords were also not merged for synonyms (e.g. ‘ACL’ and ‘anterior cruciate ligament’), which may have split related concepts across clusters in the thematic and trend analyses. Sixth, 2026 was a partial year at the time of the search, and three records published online as early access without a final publication were excluded, so the most recent literature is incompletely captured. Classification of document category and study design was performed by the authors from the title, abstract and WoS document type and involves some judgement. In addition, no formal inter-rater agreement statistic was calculated for the screening process. Finally, this analysis maps the volume, structure and themes of the literature rather than the clinical effectiveness of slope-correcting osteotomy. Clinical conclusions should not be drawn from the bibliometric findings alone.

## Conclusion

This bibliometric analysis shows that publication activity on slope-correcting osteotomy in the context of ACLR has increased markedly since 2017. The literature is concentrated in a small number of journals, institutions and authors, and is built on an interconnected intellectual base in which posterior tibial slope, the ACL and osteotomy form the central themes. The most cited work is predominantly biomechanical or retrospective clinical research, and no randomised trials were identified. An implication of this profile is that standardised indications and PTS measurement, and prospective comparative studies, are needed before the role of slope correction in routine ACLR practice can be defined.

## Supplementary Information

Below is the link to the electronic supplementary material.

Supplementary Material 1.

## Data Availability

All the processed data have been included in the manuscript, tables and figures. Any further data are available upon request to the corresponding author.

## Appendix 1: Search strategy

Database: Web of Science Core Collection. Search date: 15 March 2026. Search mode: Advanced Search. Records retrieved: 174.

#1 TS=(“tibial osteotom*” OR “high tibial osteotom*” OR “proximal tibial osteotom*” OR “closing wedge osteotom*” OR “anterior closing wedge” OR “deflexion osteotom*”).

#2 TS=(“posterior tibial slope” OR “tibial slope” OR “sagittal slope” OR “slope correction” OR “slope reduc*” OR “slope decreas*”).

#3 TS=(“anterior cruciate ligament” OR ACL OR “ACL reconstruction” OR ACLR OR “revision ACL” OR “failed ACL”).

#4 #1 AND #2 AND #3.

TS = Topic field (title, abstract, author keywords and Keywords Plus). No date, language or document-type limits were applied.
