# Supplementary material for: Bibliometric analysis of slope-correcting osteotomy in the context of anterior cruciate ligament reconstruction: key trends, contributors and themes
Source: Eur J Orthop Surg Traumatol. 2026 Oct 27;36(1):428. doi: 10.1007/s00590-026-05019-y (PMC13714038; doi:10.1007/s00590-026-05019-y)
Supplement: Supplementary file 1 [file 590_2026_5019_MOESM1_ESM.docx]

**Supplementary material**

*Bibliometric analysis of slope-correcting osteotomy in the context of anterior cruciate ligament reconstruction: key trends, contributors and themes*

## **Supplementary Table S1. The 50 most cited publications**

| **Rank** | **First author** | **Year** | **Title** | **Journal** | **Citations (WoS)** | **Annual citation rate*** | **Category** |
| --- | --- | --- | --- | --- | --- | --- | --- |
| 1 | Dejour, D | 2015 | Tibial slope correction combined with second revision ACL produces good knee stability and prevents graft rupture | Knee Surgery Sports Traumatology Arthroscopy | 238 | 19.8 | Original research – clinical |
| 2 | Shelburne, KB | 2011 | Effect of Posterior Tibial Slope on Knee Biomechanics during Functional Activity | Journal of Orthopaedic Research | 214 | 13.4 | Original research – biomechanical/cadaveric |
| 3 | Bernhardson, AS | 2019 | Tibial Slope and Its Effect on Force in Anterior Cruciate Ligament Grafts: Anterior Cruciate Ligament Force Increases Linearly as Posterior Tibial Slope Increases | American Journal of Sports Medicine | 209 | 26.1 | Original research – biomechanical/cadaveric |
| 4 | Sonnery-Cottet, B | 2014 | Proximal Tibial Anterior Closing Wedge Osteotomy in Repeat Revision of Anterior Cruciate Ligament Reconstruction | American Journal of Sports Medicine | 199 | 15.3 | Original research – clinical |
| 5 | Rodner, CM | 2006 | Medial opening wedge tibial osteotomy and the sagittal plane - The effect of increasing tibial slope on tibiofemoral contact pressure | American Journal of Sports Medicine | 148 | 7.0 | Original research – biomechanical/cadaveric |
| 6 | Imhoff, FB | 2019 | Slope-reducing tibial osteotomy decreases ACL-graft forces and anterior tibial translation under axial load | Knee Surgery Sports Traumatology Arthroscopy | 123 | 15.4 | Original research – biomechanical/cadaveric |
| 7 | Song, GY | 2020 | Slope-Reducing Tibial Osteotomy Combined With Primary Anterior Cruciate Ligament Reconstruction Produces Improved Knee Stability in Patients With Steep Posterior Tibial Slope, Excessive Anterior Tibial Subluxation in Extension, and Chronic Meniscal Posterior Horn Tears | American Journal of Sports Medicine | 90 | 12.9 | Original research – clinical |
| 8 | Yamaguchi, KT | 2018 | Effects of Anterior Closing Wedge Tibial Osteotomy on Anterior Cruciate Ligament Force and Knee Kinematics | American Journal of Sports Medicine | 80 | 8.9 | Original research – biomechanical/cadaveric |
| 9 | Tischer, T | 2017 | The Impact of Osseous Malalignment and Realignment Procedures in Knee Ligament Surgery A Systematic Review of the Clinical Evidence | Orthopaedic Journal of Sports Medicine | 70 | 7.0 | Systematic review/meta-analysis |
| 10= | Cantin, O | 2015 | The role of high tibial osteotomy in the treatment of knee laxity: a comprehensive review | Knee Surgery Sports Traumatology Arthroscopy | 65 | 5.4 | Narrative review |
| 10= | Voos, JE | 2012 | Effect of tibial slope on the stability of the anterior cruciate ligament-deficient knee | Knee Surgery Sports Traumatology Arthroscopy | 65 | 4.3 | Original research – biomechanical/cadaveric |
| 12 | Zaffagnini, S | 2013 | Combined ACL reconstruction and closing-wedge HTO for varus angulated ACL-deficient knees | Knee Surgery Sports Traumatology Arthroscopy | 64 | 4.6 | Original research – clinical |
| 13 | Imhoff, FB | 2021 | Effect of Slope and Varus Correction High Tibial Osteotomy in the ACL-Deficient and ACL-Reconstructed Knee on Kinematics and ACL Graft Force: A Biomechanical Analysis | American Journal of Sports Medicine | 63 | 10.5 | Original research – biomechanical/cadaveric |
| 14 | Ni, QK | 2020 | Steep Posterior Tibial Slope and Excessive Anterior Tibial Translation Are Predictive Risk Factors of Primary Anterior Cruciate Ligament Reconstruction Failure: A Case-Control Study With Prospectively Collected Data | American Journal of Sports Medicine | 62 | 8.9 | Original research – clinical |
| 15= | Rozinthe, A | 2022 | Tibial slope correction combined with second revision ACLR grants good clinical outcomes and prevents graft rupture at 7-15-year follow-up | Knee Surgery Sports Traumatology Arthroscopy | 51 | 10.2 | Original research – clinical |
| 15= | Dean, CS | 2016 | Clinical Outcomes of High Tibial Osteotomy for Knee Instability: A Systematic Review | Orthopaedic Journal of Sports Medicine | 51 | 4.6 | Systematic review/meta-analysis |
| 17 | Marriott, K | 2015 | Five-Year Changes in Gait Biomechanics After Concomitant High Tibial Osteotomy and ACL Reconstruction in Patients With Medial Knee Osteoarthritis | American Journal of Sports Medicine | 45 | 3.8 | Original research – clinical |
| 18= | Bosco, F | 2023 | Could anterior closed-wedge high tibial osteotomy be a viable option in patients with high posterior tibial slope who undergo anterior cruciate ligament reconstruction? A systematic review and meta-analysis | European Journal of Orthopaedic Surgery and Traumatology | 44 | 11.0 | Systematic review/meta-analysis |
| 18= | Weiler, A | 2022 | Significant slope reduction in ACL deficiency can be achieved both by anterior closing-wedge and medial open-wedge high tibial osteotomies: early experiences in 76 cases | Knee Surgery Sports Traumatology Arthroscopy | 44 | 8.8 | Original research – clinical |
| 20 | Southam, BR | 2018 | Underappreciated Factors to Consider in Revision Anterior Cruciate Ligament Reconstruction A Current Concepts Review | Orthopaedic Journal of Sports Medicine | 43 | 4.8 | Narrative review |
| 21 | Dean, RS | 2021 | Posterior Tibial Slope: Understand Bony Morphology to Protect Knee Cruciate Ligament Grafts | Arthroscopy: The Journal of Arthroscopic and Related Surgery | 39 | 6.5 | Editorial |
| 22= | Samuelsen, BT | 2020 | Posterior Medial Meniscus Root Tears Potentiate the Effect of Increased Tibial Slope on Anterior Cruciate Ligament Graft Forces | American Journal of Sports Medicine | 38 | 5.4 | Original research – biomechanical/cadaveric |
| 22= | Arun, GR | 2016 | Long-term follow up of single-stage anterior cruciate ligament reconstruction and high tibial osteotomy and its relation with posterior tibial slope | Archives of Orthopaedic and Trauma Surgery | 38 | 3.5 | Original research – clinical |
| 24= | Dan, MJ | 2024 | Four to 6° Is the Target Posterior Tibial Slope After Tibial Deflection Osteotomy According to the Knee Static Anterior Tibial Translation | Arthroscopy: The Journal of Arthroscopic and Related Surgery | 35 | 11.7 | Original research – clinical |
| 24= | Mabrouk, A | 2023 | Outcomes of Slope-Reducing Proximal Tibial Osteotomy Combined With a Third Anterior Cruciate Ligament Reconstruction Procedure With a Focus on Return to Impact Sports | American Journal of Sports Medicine | 35 | 8.8 | Original research – clinical |
| 26 | Kim, GB | 2019 | Increased Posterior Tibial Slope After Medial Open-Wedge High Tibial Osteotomy May Result in Degenerative Changes in Anterior Cruciate Ligament | Journal of Arthroplasty | 34 | 4.2 | Original research – clinical |
| 27= | Dejour, D | 2023 | First revision ACL reconstruction combined with tibial deflexion osteotomy improves clinical scores at 2 to 7 years follow-up | Knee Surgery Sports Traumatology Arthroscopy | 32 | 8.0 | Original research – clinical |
| 27= | LERAT, JL | 1993 | ANTERIOR LAXITY AND MEDIAL ARTHRITIS IN VARUS KNEES - RESULTS OF THE RECONSTRUCTION OF THE ANTERIOR CRUCIATE LIGAMENT ASSOCIATED WITH A HIGH TIBIAL OSTEOTOMY | Revue de Chirurgie Orthopédique et Réparatrice de l'Appareil Moteur | 32 | 0.9 | Original research – clinical |
| 29= | Ni, QK | 2022 | Posterior tibial slope measurements based on the full-length tibial anatomic axis are significantly increased compared to those based on the half-length tibial anatomic axis | Knee Surgery Sports Traumatology Arthroscopy | 30 | 6.0 | Original research – radiographic/imaging/planning |
| 29= | Dejour, D | 2017 | Sagittal Plane Corrections around the Knee | Journal of Knee Surgery | 30 | 3.0 | Narrative review |
| 31= | Nazzal, EM | 2022 | Considerations of the Posterior Tibial Slope in Anterior Cruciate Ligament Reconstruction: a Scoping Review | Current Reviews in Musculoskeletal Medicine | 28 | 5.6 | Narrative review |
| 31= | Ogawa, H | 2019 | Effect of increased posterior tibial slope on the anterior cruciate ligament status in medial open wedge high tibial osteotomy in an uninjured ACL population | Orthopaedics & Traumatology: Surgery & Research | 28 | 3.5 | Original research – clinical |
| 33= | Kayaalp, ME | 2024 | Around-the-knee osteotomies part 1: definitions, rationale and planning-state of the art | Journal of ISAKOS Joint Disorders & Orthopaedic Sports Medicine | 27 | 9.0 | Narrative review |
| 33= | Cantivalli, A | 2019 | High Tibial Osteotomy and Anterior Cruciate Ligament Reconstruction/Revision | Clinics in Sports Medicine | 27 | 3.4 | Narrative review |
| 35 | Martineau, PA | 2010 | Anterior opening wedge high tibial osteotomy: the effect of increasing posterior tibial slope on ligament strain | Canadian Journal of Surgery | 26 | 1.5 | Original research – biomechanical/cadaveric |
| 36= | Duerr, RA | 2023 | Association of Elevated Posterior Tibial Slope With Revision Anterior Cruciate Ligament Graft Failure in a Matched Cohort Analysis | American Journal of Sports Medicine | 24 | 6.0 | Original research – clinical |
| 36= | Jeong, SH | 2022 | Patient-specific high tibial osteotomy for varus malalignment: 3D-printed plating technique and review of the literature | European Journal of Orthopaedic Surgery and Traumatology | 24 | 4.8 | Surgical technique report |
| 38 | Lott, A | 2024 | Around-the-knee osteotomies part II: Surgical indications, techniques and outcomes - State of the art | Journal of ISAKOS Joint Disorders & Orthopaedic Sports Medicine | 22 | 7.3 | Narrative review |
| 39= | Gupta, A | 2019 | Surgical Techniques, Outcomes, Indications, and Complications of Simultaneous High Tibial Osteotomy and Anterior Cruciate Ligament Revision Surgery: A Systematic Review | HSS Journal | 21 | 2.6 | Systematic review/meta-analysis |
| 39= | Neyret, P | 2000 | Tibial deflexion osteotomy | Operative Techniques in Sports Medicine | 21 | 0.8 | Surgical technique report |
| 41 | Alaia, MJ | 2021 | Tibial Sagittal Slope in Anterior Cruciate Ligament Injury and Treatment | Journal of the American Academy of Orthopaedic Surgeons | 20 | 3.3 | Narrative review |
| 42= | Cance, N | 2024 | Radiographic Investigation of Coronal Plane and Patellar Height and Changes Following Tibial Deflection Osteotomy for Correction of Tibial Slope in Combination With ACL Reconstruction | American Journal of Sports Medicine | 19 | 6.3 | Original research – radiographic/imaging/planning |
| 42= | Diquattro, E | 2023 | ACL surgery: reasons for failure and management | EFORT Open Reviews | 19 | 4.8 | Narrative review |
| 42= | Klek, M | 2019 | The Role of High Tibial Osteotomy in ACL Reconstruction in Knees with Coronal and Sagittal Plane Deformity | Current Reviews in Musculoskeletal Medicine | 19 | 2.4 | Narrative review |
| 45 | Ranawat, AS | 2016 | Comparison of Lateral Closing-Wedge Versus Medial Opening-Wedge High Tibial Osteotomy on Knee Joint Alignment and Kinematics in the ACL-Deficient Knee | American Journal of Sports Medicine | 18 | 1.6 | Original research – biomechanical/cadaveric |
| 46= | Kayaalp, ME | 2024 | Slope Osteotomies in the Setting of Anterior Cruciate Ligament Deficiency | Journal of Bone and Joint Surgery-American Volume | 17 | 5.7 | Narrative review |
| 46= | Vadhera, AS | 2021 | Current Concepts in Anterior Tibial Closing Wedge Osteotomies for Anterior Cruciate Ligament Deficient Knees | Current Reviews in Musculoskeletal Medicine | 17 | 2.8 | Narrative review |
| 48 | Guy, S | 2024 | The Influence of Tibial Tubercle-Sparing Slope-Reducing Osteotomy on Patellar Height in Patients Undergoing Revision ACL Reconstruction | American Journal of Sports Medicine | 16 | 5.3 | Original research – clinical |
| 49= | Mayer, P | 2023 | Effect of Anterior Tibial Closing Wedge Osteotomy on Coronal Tibial Alignment in Relation to Preoperative Medial Proximal Tibial Angle and Wedge Height | American Journal of Sports Medicine | 15 | 3.8 | Original research – radiographic/imaging/planning |
| 49= | Monllau, JC | 2023 | Anterior cruciate ligament failure and management | EFORT Open Reviews | 15 | 3.8 | Narrative review |

*= denotes tied rank. *Total WoS Core Collection citations (15 March 2026) divided by the number of years from the year of publication to 2026, inclusive.*

## **Supplementary Table S2. Records excluded during selection, with reasons (n = 23)**

| **First author** | **Year** | **Title** | **Journal** | **WoS document type** | **Reason for exclusion** |
| --- | --- | --- | --- | --- | --- |
| Kim, BS | 2022 | No Differences In Clinical Outcomes Between Rectangular and Round Tunnel Techniques For Anterior Crucial Ligament Reconstruction | Arthroscopy: The Journal of Arthroscopic and Related Surgery | Article | ACL surgery not concerning PTS or osteotomy |
| Imhoff, F | 2019 | Slope-reducing tibial osteotomy decreases ACL-graft forces and anterior tibial translation under axial load | Swiss Medical Weekly | Meeting Abstract | Duplicate publication (conference abstract of an included article) |
| Dickschas, J | 2025 | Anterior Closed Wedge High Tibial Osteotomy for Slope Correction in Anterior Cruciate Ligament Insufficiency: A Preliminary Report of 100 Cases at a 2-Year Follow-up | Cartilage | Article; Early Access | Early access without final publication |
| Kayaalp, ME | 2025 | Infratuberosity osteotomies require greater wedge resection and result in increased cortical mismatch compared to supratuberosity: A morphometric study supporting individualized planning in posterior tibial slope correction | Knee Surgery Sports Traumatology Arthroscopy | Article; Early Access | Early access without final publication |
| Kunze, KN | 2026 | Hardware-related symptoms are the most common complication after anterior closing wedge osteotomy performed with ACL reconstruction: A systematic review | Knee Surgery Sports Traumatology Arthroscopy | Review; Early Access | Early access without final publication |
| Portner, O | 2014 | High Tibial Valgus Osteotomy: Closing, Opening or Combined? Patellar Height as a Determining Factor | Clinical Orthopaedics and Related Research | Article | Osteotomy or PTS studied without the ACL as a subject (e.g. HTO for osteoarthritis, genu recurvatum) |
| Kuriyama, S | 2020 | Classical target coronal alignment in high tibial osteotomy demonstrates validity in terms of knee kinematics and kinetics in a computer model | Knee Surgery Sports Traumatology Arthroscopy | Article | Osteotomy or PTS studied without the ACL as a subject (e.g. HTO for osteoarthritis, genu recurvatum) |
| Dean, RS | 2020 | Treatment for Symptomatic Genu Recurvatum: A Systematic Review | Orthopaedic Journal of Sports Medicine | Review | Osteotomy or PTS studied without the ACL as a subject (e.g. HTO for osteoarthritis, genu recurvatum) |
| Ogawa, H | 2018 | New angle measurement device to control the posterior tibial slope angle in medial opening wedge high tibial osteotomy | Archives of Orthopaedic and Trauma Surgery | Article | Osteotomy or PTS studied without the ACL as a subject (e.g. HTO for osteoarthritis, genu recurvatum) |
| Eliasberg, CD | 2022 | Extreme Hinge Axis Positions Are Necessary to Achieve Posterior Tibial Slope Reduction With Small Coronal-Plane Corrections in Medial Opening Wedge High Tibial Osteotomy | Orthopaedic Journal of Sports Medicine | Article | Osteotomy or PTS studied without the ACL as a subject (e.g. HTO for osteoarthritis, genu recurvatum) |
| Winkler, PW | 2025 | Meniscal forces and knee kinematics are affected by tibial slope modifying high tibial osteotomy | Knee Surgery Sports Traumatology Arthroscopy | Article | Osteotomy or PTS studied without the ACL as a subject (e.g. HTO for osteoarthritis, genu recurvatum) |
| Macleod, AR | 2023 | A simple formula to control posterior tibial slope during proximal tibial osteotomies | Clinical Biomechanics | Article | Osteotomy or PTS studied without the ACL as a subject (e.g. HTO for osteoarthritis, genu recurvatum) |
| Erquicia, JI | 2022 | A lower starting point for the medial cut increases the posterior slope in opening-wedge high tibial osteotomy: a cadaveric study | Journal of Experimental Orthopaedics | Article | Osteotomy or PTS studied without the ACL as a subject (e.g. HTO for osteoarthritis, genu recurvatum) |
| Clemens, B | 2022 | Knee kinematics are variously influenced by different correction angles in high tibial osteotomy (HTO) | International Orthopaedics | Article | Osteotomy or PTS studied without the ACL as a subject (e.g. HTO for osteoarthritis, genu recurvatum) |
| Chen, Y | 2022 | Radiographic measurement of the posterior tibial slope in normal Chinese adults: a retrospective cohort study | BMC Musculoskeletal Disorders | Article | PTS measurement or anatomy study without reference to osteotomy in the ACL setting |
| Vieider, RP | 2024 | Malrotated lateral knee radiographs do not allow for a proper assessment of medial or lateral posterior tibial slope | Knee Surgery Sports Traumatology Arthroscopy | Article | PTS measurement or anatomy study without reference to osteotomy in the ACL setting |
| Hassa, E | 2023 | CT-based analysis of posterior tibial slope in a Turkish population sample: A retrospective observational study | Medicine | Article | PTS measurement or anatomy study without reference to osteotomy in the ACL setting |
| Kumar, P | 2022 | Measurement of Posterior Tibial Slope in Healthy Indian Population: A CT-Based Study | Indian Journal of Orthopaedics | Article | PTS measurement or anatomy study without reference to osteotomy in the ACL setting |
| Singh, S | 2023 | Anatomical Variations in the Posterior Tibial Slope in the North Indian Population: A Hospital-Based Study | Cureus Journal of Medical Science | Article | PTS measurement or anatomy study without reference to osteotomy in the ACL setting |
| Arthur, A | 2007 | Proximal tibial opening wedge osteotomy as the initial treatment for chronic posterolateral corner deficiency in the varus knee - A prospective clinical study | American Journal of Sports Medicine | Article | Population not the ACL-deficient/reconstructed knee (PCL or posterolateral corner) |
| Petrigliano, FA | 2012 | The Effect of Proximal Tibial Slope on Dynamic Stability Testing of the Posterior Cruciate Ligament- and Posterolateral Corner-Deficient Knee | American Journal of Sports Medicine | Article; Proceedings Paper | Population not the ACL-deficient/reconstructed knee (PCL or posterolateral corner) |
| Reichwein, F | 2007 | High tibial flexion osteotomy for revision of posterior cruciate ligament instability | Unfallchirurg | Article | Population not the ACL-deficient/reconstructed knee (PCL or posterolateral corner) |
| Conyer, RT | 2024 | Effect of Posterior Tibial Slope on Outcomes After Posterior Cruciate Ligament Reconstruction | Orthopaedic Journal of Sports Medicine | Article | Population not the ACL-deficient/reconstructed knee (PCL or posterolateral corner) |

## **Supplementary Table S3. Included publications and document category (n = 151)**

| **First author** | **Year** | **Title** | **Journal** | **DOI** | **Category** |
| --- | --- | --- | --- | --- | --- |
| LERAT, JL | 1993 | ANTERIOR LAXITY AND MEDIAL ARTHRITIS IN VARUS KNEES - RESULTS OF THE RECONSTRUCTION OF THE ANTERIOR CRUCIATE LIGAMENT ASSOCIATED WITH A HIGH TIBIAL OSTEOTOMY | Revue de Chirurgie Orthopédique et Réparatrice de l'Appareil Moteur |  | Original research – clinical |
| Neyret, P | 2000 | Tibial deflexion osteotomy | Operative Techniques in Sports Medicine | 10.1016/S1060-1872(00)80027-1 | Surgical technique report |
| Martinek, V | 2003 | Tibial adjustment osteotomy at the kneejoint - renaissance of a classic procedure? | Deutsche Zeitschrift für Sportmedizin |  | Narrative review |
| Rodner, CM | 2006 | Medial opening wedge tibial osteotomy and the sagittal plane - The effect of increasing tibial slope on tibiofemoral contact pressure | American Journal of Sports Medicine | 10.1177/0363546506287297 | Original research – biomechanical/cadaveric |
| Jung, KA | 2009 | ACL injury while jumping rope in a patient with an unintended increase in the tibial slope after an opening wedge high tibial osteotomy | Archives of Orthopaedic and Trauma Surgery | 10.1007/s00402-008-0739-y | Original research – clinical |
| Martineau, PA | 2010 | Anterior opening wedge high tibial osteotomy: the effect of increasing posterior tibial slope on ligament strain | Canadian Journal of Surgery |  | Original research – biomechanical/cadaveric |
| Shelburne, KB | 2011 | Effect of Posterior Tibial Slope on Knee Biomechanics during Functional Activity | Journal of Orthopaedic Research | 10.1002/jor.21242 | Original research – biomechanical/cadaveric |
| Voos, JE | 2012 | Effect of tibial slope on the stability of the anterior cruciate ligament-deficient knee | Knee Surgery Sports Traumatology Arthroscopy | 10.1007/s00167-011-1823-3 | Original research – biomechanical/cadaveric |
| Zaffagnini, S | 2013 | Combined ACL reconstruction and closing-wedge HTO for varus angulated ACL-deficient knees | Knee Surgery Sports Traumatology Arthroscopy | 10.1007/s00167-013-2400-8 | Original research – clinical |
| Sonnery-Cottet, B | 2014 | Proximal Tibial Anterior Closing Wedge Osteotomy in Repeat Revision of Anterior Cruciate Ligament Reconstruction | American Journal of Sports Medicine | 10.1177/0363546514534938 | Original research – clinical |
| Cantin, O | 2015 | The role of high tibial osteotomy in the treatment of knee laxity: a comprehensive review | Knee Surgery Sports Traumatology Arthroscopy | 10.1007/s00167-015-3752-z | Narrative review |
| Dejour, D | 2015 | Tibial slope correction combined with second revision ACL produces good knee stability and prevents graft rupture | Knee Surgery Sports Traumatology Arthroscopy | 10.1007/s00167-015-3758-6 | Original research – clinical |
| Marriott, K | 2015 | Five-Year Changes in Gait Biomechanics After Concomitant High Tibial Osteotomy and ACL Reconstruction in Patients With Medial Knee Osteoarthritis | American Journal of Sports Medicine | 10.1177/0363546515591995 | Original research – clinical |
| Arun, GR | 2016 | Long-term follow up of single-stage anterior cruciate ligament reconstruction and high tibial osteotomy and its relation with posterior tibial slope | Archives of Orthopaedic and Trauma Surgery | 10.1007/s00402-015-2385-5 | Original research – clinical |
| Dean, CS | 2016 | Clinical Outcomes of High Tibial Osteotomy for Knee Instability: A Systematic Review | Orthopaedic Journal of Sports Medicine | 10.1177/2325967116633419 | Systematic review/meta-analysis |
| Ranawat, AS | 2016 | Comparison of Lateral Closing-Wedge Versus Medial Opening-Wedge High Tibial Osteotomy on Knee Joint Alignment and Kinematics in the ACL-Deficient Knee | American Journal of Sports Medicine | 10.1177/0363546516657824 | Original research – biomechanical/cadaveric |
| Robin, JG | 2016 | High tibial osteotomy in knee laxities: Concepts review and results | EFORT Open Reviews | 10.1302/2058-5241.1.000001 | Narrative review |
| Crawford, MD | 2017 | Surgical Management and Treatment of the Anterior Cruciate Ligament-Deficient Knee with Malalignment | Clinics in Sports Medicine | 10.1016/j.csm.2016.08.006 | Narrative review |
| Dean, CS | 2017 | Posteromedially placed plates with anterior staple reinforcement are not successful in decreasing tibial slope in opening-wedge proximal tibial osteotomy | Knee Surgery Sports Traumatology Arthroscopy | 10.1007/s00167-016-4311-y | Original research – radiographic/imaging/planning |
| Dejour, D | 2017 | Sagittal Plane Corrections around the Knee | Journal of Knee Surgery | 10.1055/s-0037-1603637 | Narrative review |
| Feucht, MJ | 2017 | Osteotomies around the knee for ligament insufficiency | Orthopäde | 10.1007/s00132-017-3439-x | Narrative review |
| Tischer, T | 2017 | The Impact of Osseous Malalignment and Realignment Procedures in Knee Ligament Surgery A Systematic Review of the Clinical Evidence | Orthopaedic Journal of Sports Medicine | 10.1177/2325967117697287 | Systematic review/meta-analysis |
| Kim, KI | 2018 | Does the pre-operative status of the anterior cruciate ligament affect the outcomes following medial open-wedge high tibial osteotomy? | Knee | 10.1016/j.knee.2018.10.010 | Original research – clinical |
| Schuster, P | 2018 | The influence of tibial slope on the graft in combined high tibial osteotomy and anterior cruciate ligament reconstruction | Knee | 10.1016/j.knee.2018.04.007 | Original research – clinical |
| Southam, BR | 2018 | Underappreciated Factors to Consider in Revision Anterior Cruciate Ligament Reconstruction A Current Concepts Review | Orthopaedic Journal of Sports Medicine | 10.1177/2325967117751689 | Narrative review |
| Yamaguchi, KT | 2018 | Effects of Anterior Closing Wedge Tibial Osteotomy on Anterior Cruciate Ligament Force and Knee Kinematics | American Journal of Sports Medicine | 10.1177/0363546517736767 | Original research – biomechanical/cadaveric |
| Bernhardson, AS | 2019 | Tibial Slope and Its Effect on Force in Anterior Cruciate Ligament Grafts: Anterior Cruciate Ligament Force Increases Linearly as Posterior Tibial Slope Increases | American Journal of Sports Medicine | 10.1177/0363546518820302 | Original research – biomechanical/cadaveric |
| Cantivalli, A | 2019 | High Tibial Osteotomy and Anterior Cruciate Ligament Reconstruction/Revision | Clinics in Sports Medicine | 10.1016/j.csm.2019.02.008 | Narrative review |
| Gupta, A | 2019 | Surgical Techniques, Outcomes, Indications, and Complications of Simultaneous High Tibial Osteotomy and Anterior Cruciate Ligament Revision Surgery: A Systematic Review | HSS Journal | 10.1007/s11420-018-9630-8 | Systematic review/meta-analysis |
| Imhoff, FB | 2019 | Slope-reducing tibial osteotomy decreases ACL-graft forces and anterior tibial translation under axial load | Knee Surgery Sports Traumatology Arthroscopy | 10.1007/s00167-019-05360-2 | Original research – biomechanical/cadaveric |
| Kim, GB | 2019 | Increased Posterior Tibial Slope After Medial Open-Wedge High Tibial Osteotomy May Result in Degenerative Changes in Anterior Cruciate Ligament | Journal of Arthroplasty | 10.1016/j.arth.2019.04.032 | Original research – clinical |
| Klek, M | 2019 | The Role of High Tibial Osteotomy in ACL Reconstruction in Knees with Coronal and Sagittal Plane Deformity | Current Reviews in Musculoskeletal Medicine | 10.1007/s12178-019-09589-9 | Narrative review |
| Kwon, OJ | 2019 | Morphologic MRI changes of the anterior cruciate ligament are associated with an increase in the medial tibial plateau bony slope after medial opening wedge high tibial osteotomy in a non-injured ACL population | Orthopaedics & Traumatology: Surgery & Research | 10.1016/j.otsr.2019.08.005 | Original research – radiographic/imaging/planning |
| Münch, L | 2019 | Update on Failure Analysis of Implants in ACL Surgery: Technical Failure or Fate? | Zeitschrift für Orthopädie und Unfallchirurgie | 10.1055/a-0774-8272 | Narrative review |
| Ogawa, H | 2019 | Effect of increased posterior tibial slope on the anterior cruciate ligament status in medial open wedge high tibial osteotomy in an uninjured ACL population | Orthopaedics & Traumatology: Surgery & Research | 10.1016/j.otsr.2019.05.003 | Original research – clinical |
| Reider, B | 2019 | Slippery Slope | American Journal of Sports Medicine | 10.1177/0363546519825850 | Editorial |
| Kim, JE | 2020 | How does geometric change after open-wedge high tibial osteotomy affect anterior cruciate ligament status? | Knee | 10.1016/j.knee.2020.04.008 | Original research – clinical |
| Ni, QK | 2020 | Steep Posterior Tibial Slope and Excessive Anterior Tibial Translation Are Predictive Risk Factors of Primary Anterior Cruciate Ligament Reconstruction Failure: A Case-Control Study With Prospectively Collected Data | American Journal of Sports Medicine | 10.1177/0363546520949212 | Original research – clinical |
| Samuelsen, BT | 2020 | Posterior Medial Meniscus Root Tears Potentiate the Effect of Increased Tibial Slope on Anterior Cruciate Ligament Graft Forces | American Journal of Sports Medicine | 10.1177/0363546519889628 | Original research – biomechanical/cadaveric |
| Song, GY | 2020 | Slope-Reducing Tibial Osteotomy Combined With Primary Anterior Cruciate Ligament Reconstruction Produces Improved Knee Stability in Patients With Steep Posterior Tibial Slope, Excessive Anterior Tibial Subluxation in Extension, and Chronic Meniscal Posterior Horn Tears | American Journal of Sports Medicine | 10.1177/0363546520963083 | Original research – clinical |
| Alaia, MJ | 2021 | Tibial Sagittal Slope in Anterior Cruciate Ligament Injury and Treatment | Journal of the American Academy of Orthopaedic Surgeons | 10.5435/JAAOS-D-21-00143 | Narrative review |
| Cruz, CA | 2021 | Tibial Tubercle-Sparing Anterior Closing Wedge Osteotomy With Cross-Screw Fixation to Correct Pathologic Posterior Tibial Slope | Arthroscopy Techniques | 10.1016/j.eats.2020.11.005 | Surgical technique report |
| Dean, RS | 2021 | Posterior Tibial Slope: Understand Bony Morphology to Protect Knee Cruciate Ligament Grafts | Arthroscopy: The Journal of Arthroscopic and Related Surgery | 10.1016/j.arthro.2021.05.006 | Editorial |
| Dean, RS | 2021 | Knee Malignment: Is There a Role for Correction in Primary ACLR? | Operative Techniques in Sports Medicine | 10.1016/j.otsm.2021.150833 | Narrative review |
| Floyd, ER | 2021 | Tibial Tubercle Preserving Anterior Closing Wedge Proximal Tibial Osteotomy and ACL Tunnel Bone Grafting for Increased Posterior Tibial Slope in Failed ACL Reconstructions | Arthroscopy Techniques | 10.1016/j.eats.2021.05.026 | Surgical technique report |
| Imhoff, FB | 2021 | Effect of Slope and Varus Correction High Tibial Osteotomy in the ACL-Deficient and ACL-Reconstructed Knee on Kinematics and ACL Graft Force: A Biomechanical Analysis | American Journal of Sports Medicine | 10.1177/0363546520976147 | Original research – biomechanical/cadaveric |
| Lucidi, GA | 2021 | Editorial Commentary: The Importance of Bony Morphology in the Anterior Cruciate Ligament-Injured Patient | Arthroscopy: The Journal of Arthroscopic and Related Surgery | 10.1016/j.arthro.2021.05.043 | Editorial |
| Martinez-Cano, JP | 2021 | Editorial Commentary: Anterolateral Hinge Position Decreases the Slope in Opening-Wedge High Tibial Osteotomy: A Key Point in Treating an Anterior Cruciate Ligament-Deficient, Varus Knee | Arthroscopy: The Journal of Arthroscopic and Related Surgery | 10.1016/j.arthro.2021.01.050 | Editorial |
| Quinn, CA | 2021 | Determining the Ratio of Wedge Height to Degree of Correction for Anterior Tibial Closing Wedge Osteotomies for Excessive Posterior Tibial Slope | American Journal of Sports Medicine | 10.1177/03635465211044136 | Original research – radiographic/imaging/planning |
| Vadhera, AS | 2021 | Current Concepts in Anterior Tibial Closing Wedge Osteotomies for Anterior Cruciate Ligament Deficient Knees | Current Reviews in Musculoskeletal Medicine | 10.1007/s12178-021-09729-0 | Narrative review |
| Zee, MJM | 2021 | The correlation between posterior tibial slope and dynamic anterior tibial translation and dynamic range of tibial rotation | Journal of Experimental Orthopaedics | 10.1186/s40634-021-00389-0 | Original research – clinical |
| Ganokroj, P | 2022 | Anterior Closing-Wedge High Tibial Slope-Correcting Osteotomy Using Patient-Specific Preoperative Planning Software for Failed Anterior Cruciate Ligament Reconstruction | Arthroscopy Techniques | 10.1016/j.eats.2022.07.015 | Surgical technique report |
| Guy, S | 2022 | Anterior Closing-Wedge High Tibial Osteotomy Using Patient-Specific Cutting Guide in Chronic Anterior Cruciate Ligament-Deficient Knees | Arthroscopy Techniques | 10.1016/j.eats.2022.05.006 | Surgical technique report |
| Iseki, T | 2022 | Increased posterior slope and coronal inclination of the tibial joint line after opening wedge high tibial osteotomy may induce mucoid degeneration of the anterior cruciate ligament: A case report | Journal of ISAKOS Joint Disorders & Orthopaedic Sports Medicine | 10.1016/j.jisako.2022.08.006 | Original research – radiographic/imaging/planning |
| Jeong, SH | 2022 | Patient-specific high tibial osteotomy for varus malalignment: 3D-printed plating technique and review of the literature | European Journal of Orthopaedic Surgery and Traumatology | 10.1007/s00590-021-03043-8 | Surgical technique report |
| Koga, H | 2022 | Simultaneous Correction of Varus Deformity and Posterior Tibial Slope by Modified Hybrid Closed-Wedge High Tibial Osteotomy | Arthroscopy Techniques | 10.1016/j.eats.2022.08.007 | Surgical technique report |
| Linke, P | 2022 | Role of corrective osteotomy in chronic peripheral instability of the knee joint | Arthroskopie | 10.1007/s00142-022-00567-x | Narrative review |
| MacLean, IS | 2022 | Varus collapse following anterior closing wedge proximal tibial osteotomy for ACL revision reconstruction: a case series | Journal of Experimental Orthopaedics | 10.1186/s40634-022-00539-y | Original research – clinical |
| Mascarenhas, R | 2022 | Editorial Commentary: Osseous Anatomy of the Knee in Female Patients is a Significant Risk Factor for Anterior Cruciate Ligament Injury and Anterior Cruciate Ligament Graft Failure | Arthroscopy: The Journal of Arthroscopic and Related Surgery | 10.1016/j.arthro.2021.11.042 | Editorial |
| Nazzal, EM | 2022 | Considerations of the Posterior Tibial Slope in Anterior Cruciate Ligament Reconstruction: a Scoping Review | Current Reviews in Musculoskeletal Medicine | 10.1007/s12178-022-09767-2 | Narrative review |
| Nelson, JA | 2022 | Tibial Supra-tubercular Deflexion Osteotomy in the Management of Failed Anterior Cruciate Ligament Reconstruction: A Surgical Technique | Arthroscopy Techniques | 10.1016/j.eats.2022.03.032 | Surgical technique report |
| Ni, QK | 2022 | Posterior tibial slope measurements based on the full-length tibial anatomic axis are significantly increased compared to those based on the half-length tibial anatomic axis | Knee Surgery Sports Traumatology Arthroscopy | 10.1007/s00167-021-06605-9 | Original research – radiographic/imaging/planning |
| Rozinthe, A | 2022 | Tibial slope correction combined with second revision ACLR grants good clinical outcomes and prevents graft rupture at 7-15-year follow-up | Knee Surgery Sports Traumatology Arthroscopy | 10.1007/s00167-021-06750-1 | Original research – clinical |
| Weiler, A | 2022 | Significant slope reduction in ACL deficiency can be achieved both by anterior closing-wedge and medial open-wedge high tibial osteotomies: early experiences in 76 cases | Knee Surgery Sports Traumatology Arthroscopy | 10.1007/s00167-022-06861-3 | Original research – clinical |
| Zsidai, B | 2022 | Slope-Reducing High Tibial Osteotomy and Over-The-Top Anterior Cruciate Ligament Reconstruction With Achilles Tendon Allograft in Multiple Failed Anterior Cruciate Ligament Reconstruction | Arthroscopy Techniques | 10.1016/j.eats.2022.07.019 | Surgical technique report |
| Baker, HP | 2023 | New Considerations in ACL Surgery When Is Anatomic Reconstruction Not Enough? | Journal of Bone and Joint Surgery-American Volume | 10.2106/JBJS.22.01079 | Narrative review |
| Bosco, F | 2023 | Could anterior closed-wedge high tibial osteotomy be a viable option in patients with high posterior tibial slope who undergo anterior cruciate ligament reconstruction? A systematic review and meta-analysis | European Journal of Orthopaedic Surgery and Traumatology | 10.1007/s00590-022-03419-4 | Systematic review/meta-analysis |
| Dejour, D | 2023 | First revision ACL reconstruction combined with tibial deflexion osteotomy improves clinical scores at 2 to 7 years follow-up | Knee Surgery Sports Traumatology Arthroscopy | 10.1007/s00167-023-07493-x | Original research – clinical |
| Demey, G | 2023 | Sufficient Metaphyseal Bone for Wedge Removal and Fixation Hardware During Supratuberosity Tibial Deflexion Osteotomy in Knees With Excessive Posterior Tibial Slope | American Journal of Sports Medicine | 10.1177/03635465231175879 | Original research – radiographic/imaging/planning |
| Demey, G | 2023 | Posterior tibial slope correlated with metaphyseal inclination more than metaphyseal height | Knee | 10.1016/j.knee.2023.08.007 | Original research – radiographic/imaging/planning |
| Diquattro, E | 2023 | ACL surgery: reasons for failure and management | EFORT Open Reviews | 10.1530/EOR-23-0085 | Narrative review |
| Duerr, RA | 2023 | Association of Elevated Posterior Tibial Slope With Revision Anterior Cruciate Ligament Graft Failure in a Matched Cohort Analysis | American Journal of Sports Medicine | 10.1177/03635465221134806 | Original research – clinical |
| Imhoff, FB | 2023 | Slope and frontal axis: three-dimensional analysis and correction with patient-specific cutting guides for the proximal tibia | Operative Orthopädie und Traumatologie | 10.1007/s00064-023-00815-9 | Surgical technique report |
| Itthipanichpong, T | 2023 | Systematic Review of Clinical Outcomes After Proximal Tibia Anterior Closing-Wedge Osteotomy With ACL Reconstruction | Orthopaedic Journal of Sports Medicine | 10.1177/23259671231210549 | Systematic review/meta-analysis |
| Kim, JS | 2023 | Is there any benefit in the combined ligament reconstruction with osteotomy compared to ligament reconstruction or osteotomy alone?: Comparative outcome analysis according to the degree of medial compartment osteoarthritis with anterior or posterior cruciate ligament insufficiency | Archives of Orthopaedic and Trauma Surgery | 10.1007/s00402-022-04544-9 | Original research – clinical |
| Kraeutler, MJ | 2023 | The Principles of Knee Joint Preservation: Operative Treatment Strategies | Journal of Bone and Joint Surgery-American Volume | 10.2106/JBJS.23.00212 | Narrative review |
| Liu, JC | 2023 | Anterior closing wedge tibial osteotomy for the correction of steep posterior tibial slope and varus knee deformity associated with primary anterior cruciate ligament injury | Asian Journal of Surgery | 10.1016/j.asjsur.2023.01.016 | Letter |
| Mabrouk, A | 2023 | Outcomes of Slope-Reducing Proximal Tibial Osteotomy Combined With a Third Anterior Cruciate Ligament Reconstruction Procedure With a Focus on Return to Impact Sports | American Journal of Sports Medicine | 10.1177/03635465231203016 | Original research – clinical |
| Mayer, P | 2023 | Effect of Anterior Tibial Closing Wedge Osteotomy on Coronal Tibial Alignment in Relation to Preoperative Medial Proximal Tibial Angle and Wedge Height | American Journal of Sports Medicine | 10.1177/03635465231181553 | Original research – radiographic/imaging/planning |
| Mhaskar, VA | 2023 | Trapezoidal Wedge Osteotomy with Ligament Reconstruction for Chronic Instability following Failed ACLR | Indian Journal of Orthopaedics | 10.1007/s43465-023-00954-y | Original research – clinical |
| Monllau, JC | 2023 | Anterior cruciate ligament failure and management | EFORT Open Reviews | 10.1530/EOR-23-0037 | Narrative review |
| Müller, S | 2023 | Biplanar high tibial osteotomy for the combined correction of varus and posterior tibial slope malalignment | Orthopaedics & Traumatology: Surgery & Research | 10.1016/j.otsr.2022.103339 | Surgical technique report |
| Rassat, R | 2023 | Slope-decreasing anterior closing wedge proximal tibial osteotomies using the freehand technique are accurate to within 2° | Orthopaedics & Traumatology: Surgery & Research | 10.1016/j.otsr.2022.103466 | Original research – radiographic/imaging/planning |
| Takahashi, T | 2023 | Excellent short-term results of dome-shaped high tibial osteotomy combined with all-inside anterior cruciate ligament reconstruction | Journal of Experimental Orthopaedics | 10.1186/s40634-023-00632-w | Original research – clinical |
| Winkler, PW | 2023 | Increasing the posterior tibial slope lowers in situ forces in the native ACL primarily at deep flexion angles | Journal of Orthopaedic Research | 10.1002/jor.25484 | Original research – biomechanical/cadaveric |
| Yang, CZ | 2023 | In slope-changing osteotomy one millimeter is not one degree: results of an artificial intelligence-automated software analysis | International Orthopaedics | 10.1007/s00264-022-05676-8 | Original research – radiographic/imaging/planning |
| Baker, HP | 2024 | Minimally Invasive Anterior Closing-wedge Osteotomy for Posterior Tibial Slope Correction: A Pilot Study | Techniques in Orthopaedics | 10.1097/BTO.0000000000000658 | Original research – biomechanical/cadaveric |
| Bouché, PA | 2024 | Resurgence of slope osteotomies: A new chapter in anterior cruciate ligament surgery? | Orthopaedics & Traumatology: Surgery & Research | 10.1016/j.otsr.2024.103902 | Editorial |
| Cance, N | 2024 | Radiographic Investigation of Coronal Plane and Patellar Height and Changes Following Tibial Deflection Osteotomy for Correction of Tibial Slope in Combination With ACL Reconstruction | American Journal of Sports Medicine | 10.1177/03635465231222643 | Original research – radiographic/imaging/planning |
| Dan, MJ | 2024 | Four to 6° Is the Target Posterior Tibial Slope After Tibial Deflection Osteotomy According to the Knee Static Anterior Tibial Translation | Arthroscopy: The Journal of Arthroscopic and Related Surgery | 10.1016/j.arthro.2023.07.007 | Original research – clinical |
| Dan, MJ | 2024 | Anterior cruciate ligament zoobiquity: Can man's best friend tell us we are being too cautious with the implementation of osteotomy to correct posterior tibial slope | Knee Surgery Sports Traumatology Arthroscopy | 10.1002/ksa.12109 | Editorial |
| Demey, G | 2024 | A Supratuberosity Anterior Closing-Wedge Proximal Tibial Osteotomy Increases Patellar Height: A Simulated Time Zero Uniplanar Radiographic Study | Arthroscopy: The Journal of Arthroscopic and Related Surgery | 10.1016/j.arthro.2023.09.002 | Original research – radiographic/imaging/planning |
| Guarino, A | 2024 | The Original Technique for Tibial Deflexion Osteotomy During Revision Anterior Cruciate Ligament Reconstruction: Surgical Technique | Arthroscopy Techniques | 10.1016/j.eats.2023.08.029 | Surgical technique report |
| Guy, S | 2024 | The Influence of Tibial Tubercle-Sparing Slope-Reducing Osteotomy on Patellar Height in Patients Undergoing Revision ACL Reconstruction | American Journal of Sports Medicine | 10.1177/03635465241228264 | Original research – clinical |
| Kaarre, J | 2024 | The effect of bony morphology on anterior cruciate ligament injury and surgery | Acta Orthopaedica et Traumatologica Turcica | 10.5152/j.aott.2024.24005 | Narrative review |
| Kayaalp, ME | 2024 | Around-the-knee osteotomies part 1: definitions, rationale and planning-state of the art | Journal of ISAKOS Joint Disorders & Orthopaedic Sports Medicine | 10.1016/j.jisako.2024.02.017 | Narrative review |
| Kayaalp, ME | 2024 | Slope Osteotomies in the Setting of Anterior Cruciate Ligament Deficiency | Journal of Bone and Joint Surgery-American Volume | 10.2106/JBJS.23.01352 | Narrative review |
| Kemler, B | 2024 | Considerations for revision anterior cruciate ligament reconstruction: A review of the current literature | Journal of Orthopaedics | 10.1016/j.jor.2024.05.012 | Narrative review |
| Leluc, J | 2024 | Patient-specific cutting guides allow 1° precision in asymmetric anterior closing-wedge osteotomy | Journal of Experimental Orthopaedics | 10.1002/jeo2.70131 | Original research – radiographic/imaging/planning |
| Lott, A | 2024 | Around-the-knee osteotomies part II: Surgical indications, techniques and outcomes - State of the art | Journal of ISAKOS Joint Disorders & Orthopaedic Sports Medicine | 10.1016/j.jisako.2024.04.002 | Narrative review |
| Mabrouk, A | 2024 | Medial posterior tibial slope measurements are overestimated on long radiographs and 3D CT compared to measurements on short lateral radiographs | Journal of Experimental Orthopaedics | 10.1002/jeo2.70120 | Original research – radiographic/imaging/planning |
| Moran, TE | 2024 | Combined tibial deflexion osteotomy and anterior cruciate ligament reconstruction improves knee function and stability: A systematic review | Journal of ISAKOS Joint Disorders & Orthopaedic Sports Medicine | 10.1016/j.jisako.2024.06.010 | Systematic review/meta-analysis |
| Onishi, S | 2024 | Infratubercle Anterior Closing Wedge Osteotomy Corrects Sagittal Alignment without Affecting Coronal Alignment or Patellar Height | Journal of Clinical Medicine | 10.3390/jcm13164715 | Original research – clinical |
| Peez, C | 2024 | Anterior Slope-Modifying Osteotomies Alter the Length Change Behavior of the Superficial Medial Collateral Ligament: A Biomechanical Study | American Journal of Sports Medicine | 10.1177/03635465241280985 | Original research – biomechanical/cadaveric |
| Quinn, M | 2024 | AN UPDATE ON EMERGING TECHNIQUES AND CONSIDERATIONS IN REVISION ANTERIOR CRUCIATE LIGAMENT RECONSTRUCTION | JBJS Reviews | 10.2106/JBJS.RVW.24.00047 | Narrative review |
| Rodriguez-Merchan, C | 2024 | Simultaneous High Tibial Osteotomy and Anterior Cruciate Ligament Reconstruction in knees with Bony Frontal and/or Sagittal Deformities: Review of the Current Literature | Archives of Bone and Joint Surgery | 10.22038/ABJS.2024.78440.3610 | Narrative review |
| Servant, C | 2024 | Editorial Commentary: Different Levels for Posterior Tibial Slope Reducing Anterior Closing Wedge Osteotomy to Reduce Anterior Cruciate Ligament Reconstruction Failure Present Different Risks | Arthroscopy: The Journal of Arthroscopic and Related Surgery | 10.1016/j.arthro.2023.10.033 | Editorial |
| Siegel, MG | 2024 | Editorial Commentary: When Performing Proximal Tibial Slope Decreasing Osteotomies in Patients Undergoing Primary or Revision Anterior Cruciate Ligament Reconstruction, Surgeons Must Be Cautious to Avoid Overcorrection of the Posterior Tibial Slope: Teeter-Totter Tibial Translation | Arthroscopy: The Journal of Arthroscopic and Related Surgery | 10.1016/j.arthro.2023.07.024 | Editorial |
| Stone, JA | 2024 | The role of anterolateral complex surgery and slope-reducing osteotomies in revision ACL reconstructions: a narrative review | Annals of Joint | 10.21037/aoj-24-30 | Narrative review |
| Tollefson, LV | 2024 | Supratubercle Anterior Closing Wedge Osteotomy: No Changes in Patellar Height and Significant Decreases in Anterior Tibial Translation at 6 Months Postoperatively | American Journal of Sports Medicine | 10.1177/03635465241252982 | Original research – clinical |
| Tollefson, LV | 2024 | Concomitant Opening-Wedge Distal Femoral Osteotomy and Anterior Closing-Wedge Proximal Tibial Osteotomy With Tunnel Bone Grafting for the First Stage of a Revision Anterior Cruciate Ligament Reconstruction | Arthroscopy Techniques | 10.1016/j.eats.2024.103054 | Surgical technique report |
| Weiler, A | 2024 | Osteotomy and knee ligament instability | Arthroskopie | 10.1007/s00142-024-00703-9 | Narrative review |
| Zhao, WC | 2024 | The application of proximal tibial anterior closing wedge osteotomy in anterior cruciate ligament reconstruction | Asia-Pacific Journal of Sport Medicine Arthroscopy Rehabilitation and Technology | 10.1016/j.asmart.2024.06.001 | Original research – clinical |
| Abreu, R | 2025 | Tibial Infratubercle Anterior Closing-Wedge Slope-Reducing Osteotomy With Combined Revision Anterior Cruciate Ligament Reconstruction | Arthroscopy Techniques | 10.1016/j.eats.2025.103890 | Surgical technique report |
| Amirtharaj, MJ | 2025 | Sagittal Slope-Reducing High Tibial Osteotomy Decreases Anterior Cruciate Ligament Force and Coupled Internal Tibial Rotation Under Pivoting Loads: A Computational Modeling Study | American Journal of Sports Medicine | 10.1177/03635465251334649 | Original research – biomechanical/cadaveric |
| Arras, C | 2025 | The use of four K-wires does not lead to a reduction of the MPTA in the context of a one-dimensional tibial deflection correction of patients with ACL re-rupture and pathologically increased tibial slope | Journal of Experimental Orthopaedics | 10.1002/jeo2.70145 | Original research – radiographic/imaging/planning |
| Bernard, C | 2025 | Editorial Commentary: Measuring Posterior Tibial Slope: You Don't Always Have to Obtain a Full-Length Tibial Radiograph, but You Should Definitely Include at Least 15 cm of Proximal Tibia on a Lateral Knee Radiograph | Arthroscopy: The Journal of Arthroscopic and Related Surgery | 10.1016/j.arthro.2025.08.020 | Editorial |
| Dan, MJ | 2025 | A Radiographic Investigation Exploring Differences in Static Anterior Tibial Translation Expressed as a Percentage Between ACL-Deficient and -Intact Patients to Improve Interobserver Variability Between Different Centers | Orthopaedic Journal of Sports Medicine | 10.1177/23259671251330310 | Original research – radiographic/imaging/planning |
| Daniel, AV | 2025 | Editorial Commentary: Various High Tibial Osteotomy Techniques Show High Survivorship, Medial Opening-Wedge Technique Has Risks, and Patient-Specific Instrumentation Shows Promise | Arthroscopy: The Journal of Arthroscopic and Related Surgery | 10.1016/j.arthro.2025.05.004 | Editorial |
| Dejour, DH | 2025 | Editorial Commentary: Posterior Tibial Slope-Reducing Osteotomy Should Be Considered in Patients Having Primary Anterior Cruciate Ligament Reconstruction if Posterior Tibial Slope Is Greater Than 12° to 14° | Arthroscopy: The Journal of Arthroscopic and Related Surgery | 10.1016/j.arthro.2025.03.004 | Editorial |
| Dracic, A | 2025 | Cut-off value for the posterior tibial slope indicating the risk for retear of the anterior cruciate ligament | Knee Surgery Sports Traumatology Arthroscopy | 10.1002/ksa.12552 | Original research – clinical |
| Fink, C | 2025 | Rupture of the anterior cruciate ligament. What must be born in mind in the reconstruction? | Unfallchirurgie | 10.1007/s00113-025-01551-4 | Narrative review |
| Fritsch, L | 2025 | Slope reducing high tibial osteotomy and revision anterior cruciate ligament reconstruction leads to satisfying clinical results and a low failure rate | Journal of Experimental Orthopaedics | 10.1002/jeo2.70260 | Original research – clinical |
| Fritz, J | 2025 | Combined revision ACL reconstruction with slope-correction osteotomy and lateral extra-articular tenodesis improves stability in patients with high posterior tibial slope and pivot shift | Journal of Experimental Orthopaedics | 10.1002/jeo2.70384 | Original research – clinical |
| Jefferies, JG | 2025 | Robotically Assisted Anterior Closing Wedge High Tibial Osteotomy: A Surgical Technique Proof of Concept | Arthroscopy Techniques | 10.1016/j.eats.2025.103850 | Surgical technique report |
| Khilnani, TK | 2025 | Combined All-Inside Anterior Cruciate Ligament Reconstruction and Tibial Anterior Closing Wedge Tibial Osteotomy Using Staple Fixation: Surgical Technique | Arthroscopy Techniques | 10.1016/j.eats.2024.103262 | Surgical technique report |
| Kim, Y | 2025 | In proximal tibial anterior closing wedge (slope changing) osteotomy lower starting points imply larger bone resection | Orthopaedics & Traumatology: Surgery & Research | 10.1016/j.otsr.2024.103979 | Original research – radiographic/imaging/planning |
| Lambrey, PJ | 2025 | Long-term Outcomes of ACL Reconstruction and Posterior Tibial Slope Correction: A 20-Year Retrospective Analysis | American Journal of Sports Medicine | 10.1177/03635465251358487 | Original research – clinical |
| Lee, MG | 2025 | Editorial Commentary: Patients With Anterior Cruciate Ligament Tears and High Posterior Tibial Slope Show Increased Risk of Failure and Meniscal Root Tears | Arthroscopy: The Journal of Arthroscopic and Related Surgery | 10.1016/j.arthro.2025.04.013 | Editorial |
| Mayer, P | 2025 | Effect of Infratuberosity Anterior Closing Wedge Osteotomy for Posterior Tibial Slope Correction on Patellar Height in Patients Undergoing Revision ACL Reconstruction | American Journal of Sports Medicine | 10.1177/03635465251323623 | Original research – clinical |
| Ollivier, M | 2025 | Infratuberosity Anterior Closing-Wedge High Tibial Osteotomy for Slope Correction in Anterior Cruciate Ligament-Deficient Knees | Arthroscopy Techniques | 10.1016/j.eats.2024.103153 | Surgical technique report |
| Ollivier, M | 2025 | Slope-Reducing Tibial Plateau-Leveling Osteotomy | Arthroscopy Techniques | 10.1016/j.eats.2024.103264 | Surgical technique report |
| Ollivier, M | 2025 | Triplanar Anteromedial Closing Wedge Tibial Osteotomy in Excessive Slope and Tibial Valgum Patients | Arthroscopy Techniques | 10.1016/j.eats.2024.103400 | Surgical technique report |
| Patel, R | 2025 | Higher precision and preservation of frontal plane alignment in slope-reducing infra-tubercle compared to retro-tubercle high tibial osteotomy | Knee Surgery Sports Traumatology Arthroscopy | 10.1002/ksa.70028 | Original research – radiographic/imaging/planning |
| Petersen, W | 2025 | Indication and treatment of pathological tibial slope | Arthroskopie | 10.1007/s00142-025-00793-z | Narrative review |
| Rocca, MS | 2025 | Joint preservation procedures: osteotomies about the knee | Annals of Joint | 10.21037/aoj-24-68 | Narrative review |
| Schuster, P | 2025 | Infratuberositary slope-decreasing anterior closed wedge proximal tibial osteotomy is safe and shows rapid bone healing | Knee Surgery Sports Traumatology Arthroscopy | 10.1002/ksa.12559 | Original research – clinical |
| Shoemaker, EP | 2025 | Slope-Reducing Anterior Closing Wedge Proximal Tibial Osteotomy, Anterior Cruciate Ligament Tunnel Bone Grafting, and Unstable Medial Meniscus Ramp Repair as a First-Stage Procedure | Arthroscopy Techniques | 10.1016/j.eats.2025.103584 | Editorial |
| Shoemaker, EP | 2025 | Single-Stage Primary Anterior Cruciate Ligament Reconstruction With Slope-Reducing Anterior Closing-Wedge Proximal Tibial Osteotomy and a Lateral Extra-Articular Tenodesis | Arthroscopy Techniques | 10.1016/j.eats.2025.103674 | Surgical technique report |
| Tollefson, LV | 2025 | Slope-Reducing Proximal Tibial Osteotomy Improves Outcomes in Anterior Cruciate Ligament Reconstruction Patients With Elevated Posterior Tibial Slope, Especially Revisions and Posterior Tibial Slope ≥12 | Arthroscopy: The Journal of Arthroscopic and Related Surgery | 10.1016/j.arthro.2024.10.048 | Systematic review/meta-analysis |
| Wackerle, AM | 2025 | Assessment and surgical correction of posterior tibial slope in revision anterior cruciate ligament surgery: An international expert Delphi consensus statement | Journal of ISAKOS Joint Disorders & Orthopaedic Sports Medicine | 10.1016/j.jisako.2025.100900 | Original research – clinical |
| Wang, DF | 2025 | Effect of Slope-Reducing Tibial Osteotomy With Primary Anterior Cruciate Ligament Reconstruction on Clinical and Radiological Results in Patients With a Steep Posterior Tibial Slope and Excessive Anterior Tibial Subluxation: Propensity Score Matching With a Minimum 2-Year Follow-up | American Journal of Sports Medicine | 10.1177/03635465251330976 | Original research – clinical |
| Watrinet, J | 2025 | Axial rotation of the hinge axis can cause changes in coronal tibial alignment in anterior tibial closing wedge osteotomy in a 3D simulation model | Journal of Experimental Orthopaedics | 10.1002/jeo2.70198 | Original research – radiographic/imaging/planning |
| Yang, HY | 2025 | Development of Mucoid Degeneration of the Anterior Cruciate Ligament Is Associated With Intermediate-Term Outcomes After High Tibial Osteotomy: A Propensity Score-Matched Analysis | Orthopaedic Journal of Sports Medicine | 10.1177/23259671251391720 | Original research – clinical |
| Zhu, TW | 2025 | Revision anterior cruciate ligament reconstruction and additional surgeries: A review | Medicine | 10.1097/MD.0000000000042620 | Narrative review |
| Guarino, A | 2026 | Anterior Closing Wedge Osteotomies for Anterior Cruciate Ligament Deficiency Indications, Technique, and Outcomes | Clinics in Sports Medicine | 10.1016/j.csm.2025.01.007 | Narrative review |
| Patel, R | 2026 | Infra-tubercle osteotomy preserves coronal alignment and reduces anterior laxity compared to retro-tubercle technique in revision anterior cruciate ligament reconstruction with slope correction | Knee Surgery Sports Traumatology Arthroscopy | 10.1002/ksa.70250 | Original research – clinical |
| Ramadani, P | 2026 | Evaluation of the anterior cruciate ligament-injured high-level athlete: a narrative review | Annals of Joint | 10.21037/aoj-25-40 | Narrative review |
| Wang, DF | 2026 | Slope-reducing tibial osteotomy combined with primary or revision ACL reconstruction improves knee stability and subjective function in patients with steep posterior tibial slope: a systematic review and meta-analysis | International Journal of Surgery | 10.1097/JS9.0000000000003507 | Systematic review/meta-analysis |
| Wei, LS | 2026 | Slope-reducing tibial osteotomy combined with primary or revision ACL reconstruction improves knee stability and subjective function in patients with steep posterior tibial slope: a systematic review and meta-analysis - Correspondence | International Journal of Surgery | 10.1097/JS9.0000000000003973 | Letter |
| Whicker, EA | 2026 | Does the Medial or Lateral Tibial Slope Have a Greater Effect on ACL Reconstruction Mechanics? A Biomechanical Analysis | Orthopaedic Journal of Sports Medicine | 10.1177/23259671251405432 | Original research – biomechanical/cadaveric |

## **Supplementary Figure S1. Thematic map restricted to original research articles (n = 73)**

*Author keywords; 250 words, minimum cluster frequency 5, three labels per cluster, Walktrap clustering (bibliometrix 5.2.1), identical to the parameters used for Figure 4. The map was generated before one record was reclassified from original research to narrative review; that record’s three author keywords occur only in that record and fall below the minimum cluster frequency, so the displayed clusters are unaffected.*
